# Chiral symmetry breaking and information accumulation in pre-biological protocell evolution

**DOI:** 10.1038/s41598-025-97319-2

**Published:** 2025-04-14

**Authors:** Konstantin K. Konstantinov, Alisa F. Konstantinova

**Affiliations:** Softellect Systems, Inc., 414-300 Ave des Sommets, Verdun, QC H3E 2B7 Canada

**Keywords:** Molecular evolution, Chiral symmetry breaking, Biological information storage, Origin of life, Evolutionary theory

## Abstract

We study a linear evolutionary model based on the two-dimensional distribution of protocells by total enantiomeric excess and the amount of stored information, which they can pass from generation to generation, and without any mutual inhibition. We show that the evolution of such systems occurs in four distinct stages. The first stage is an exponential growth of the concentration of protocells near the point $\:\left(0,\:0\right)$ and it should take negligible time on a geological scale. The second stage is a diffusion-like process in both dimensions. This process can also be accompanied by a drift in the direction of increased information passed from generation to generation, provided that the appropriate linear coefficient in the information storage subspace is large enough. The third stage is a rapid symmetry breaking and formation of the species near $\:+1$ value of enantiomeric excess (assuming a small positive global enantiomeric asymmetry factor). The fourth stage is a relaxation toward a global stationary point, which is a narrow peak located near $\:+1$ value of enantiomeric excess and some optimal value of the amount of stored information.

**Supplementary Information:**

The online version contains supplementary material available at 10.1038/s41598-025-97319-2.

## Introduction

The emergence of life on Earth is a question that has captivated scientists, philosophers, and thinkers for centuries. Among the critical stages in the origins of life is the formation and evolution of protocells, the rudimentary biological compartments that preceded modern cells. While theories surrounding abiogenesis and the RNA world have garnered significant attention, the focus on protocells provides a lens through which to understand the specific transitions that must have occurred for inanimate molecules to give rise to living, self-replicating systems. This article aims to consider two highly significant facets of protocell evolution: chiral symmetry breaking and the increase in informational complexity passed down through generations. Both aspects are crucially influenced by thermodynamics, specifically by the availability of low entropy energy inflow (e.g., energy from the Sun) and the ability to dissipate extra entropy.

Chiral molecules are those that exist in non-superimposable mirror image forms, known as enantiomers. In modern biology, only one type of organic enantiomer—either the “left-handed” (L) or the “right-handed” (D) version—is overwhelmingly favored for each class of biomolecules like amino acids and sugars. This phenomenon, known as “homochirality” presents an intriguing puzzle: how did the early Earth, presumably awash in a racemic mixture of both L- and D-enantiomers, give rise to life that prefers one type over the other? The breaking of chiral symmetry, wherein one enantiomer is favored over its mirror image, is thought to be a crucial milestone in the transition from chemistry to biology^1–13^.

Related to the question of chiral symmetry breaking is the requirement for an increase in informational complexity. For life to evolve, each successive generation of protocells must not only replicate but also adapt to its environment and optimize its performance to persist. Thus, the process would involve an increased amount of information being passed from one generation to the next, allowing for the emergence of more complex structures and functions^14–16^. This increase in inherited complexity serves as a cornerstone for Darwinian evolution and eventually leads to the complex web of life as we know it today^17^.

However, neither chiral symmetry breaking nor the increase in inherited complexity can occur in a thermodynamic vacuum. Life is an inherently non-equilibrium process, requiring a constant influx of low entropy energy to maintain its ordered state^14,18,19^. This energy source, typically in the form of sunlight for modern biology, is crucial for driving the endothermic reactions required for life. Moreover, the ability of a system to dissipate the extra entropy it generates – by exporting it to its surroundings – is a defining characteristic of living systems and likely played a vital role in the emergence and evolution of protocells^14,20–22^.

This article aims to expand upon the foundational work in protocell evolution, particularly focusing on chiral symmetry breaking and increasing informational complexity as pivotal milestones. Drawing inspiration from the one-dimensional model considered by^23^, which considered the distribution of protocells based on enantiomeric excess, we extend this model into a two-dimensional framework. The extended model not only accounts for distribution by enantiomeric excess but also incorporates another crucial dimension—the amount of information inherited from one generation to the next.

The move to a two-dimensional model aims to encapsulate a more comprehensive picture of the evolutionary dynamics at play in early life forms. By considering both the chiral composition and inherited information, we hope to explore the interplay between these two essential facets and their combined influence on protocell survival and evolution. This dual consideration is consistent with the broader understanding that these processes are governed by thermodynamic factors, specifically the need for low entropy energy sources and mechanisms for effective dissipation of extra entropy^21,22^.

While previous models have often focused on either the chiral aspects^7,13^ or information complexity^15,16^, rarely have both dimensions been considered simultaneously. This article aims to bridge that gap and offer a more synthesized view of how these critical factors co-evolved in shaping the first rudimentary biological systems on Earth.

It is also known that modern life utilizes various error correction mechanisms^24^, which allow correcting replication errors. In this work we do not want to take that into account, as that ability requires some advanced cellular machinery, which could not exist before life.

## Evolution of protocells in two-dimensional space

The model considered here is essentially the model considered by^23^ extended further into two-dimensional space of chiral composition (total enantiomeric excess of the protocell) and inherited information (the amount of information passed from generation to generation). Apart from finding the stationary points, we also wanted to solve the dynamic evolution problem because it is expected to be much more complex and richer than in one-dimensional space.

As the number of organic molecules on prebiotic Earth was very large, we first start from a continuous representation of chemical systems, where the evolution of the system can be described using differential (or in this case integrodifferential) equations. This allows us to use differential and integral machinery, which, in turn allows us to make some interesting conclusions without solving the equations. However, once we get to a time evolution, the continuous representation breaks down and results in some non-existent artefacts. We will consider that in detail below when we talk about solution method.

The exact chemical reactions, which occurred in protocells on a prebiotic Earth are not known yet, and therefore we would like to concentrate on mathematical aspects of protocell evolution, rather than to speculate on what chemical reactions could have been a driving force at that time. One of the converging ideas is that the transition from non-living matter to living matter is largely due to so called pre-biological compartmentalization^25^, of which the transition from zero to just one compartment is the first crucial step. It is those, as they are called in^25^, “self-propagating, chemically simple compartmentalized mainly organic systems” that we are after in this research but without trying to get into a discussion about the unknown chemical reactions of that time. From that point of view, a protocell, which we would like to consider here, is a simple, single-compartment unit with at least some reactions inside a compartment driven by an external energy source. This single-compartment protocell requires neither a symmetry breaking to occur first, nor an advanced replication machinery as we have in modern life. Rather, a set of coupled reactions, which under the energy inflow, could increase the amounts of its reagents is what’s sufficient to start the process. A relatively simple experiment showcasing such compartmentalization was performed back in 1963^26^ and then repeated recently^27^.

Subsequently, we would like to consider a protocell as a black box, which consumes some “food” and energy out of the environment and creates another protocell. As protocells are not alive yet, we also would like to ignore the consumption of food to support a “life” of a protocell, because there is no life yet. A protocell is a relatively complex structure consisting of many molecules and so it needs more than one molecule of food to replicate. Nevertheless, it is convenient to express the models in protocells, rather than in the number of food molecules. The rate of collision between a protocell and a “molecule” of food is proportional to the concentration of protocells and a concentration of food. And food molecules are consumed one by one, not all at a time. Therefore, the reaction rate should be proportional to the first power of concentration of food and the first power of concentration of protocell. And the fact that many molecules of food are needed to replicate a protocell simply results in a replication time proportional to the number of needed molecules of food. That new protocell may have some small random mutations in comparison to the original protocell. This can be expressed as:

1 $$\:X+U\to\:U+U{\prime\:}\:$$

where $\:X$ is an amount of food “molecules”, $\:U$ is original protocell, $\:U{\prime\:}$ is a new protocell. We shall stress that $\:X$ here is a concentration of food molecules expressed in protocells, that is the actual concentration of food molecules divided by a number of food molecules needed to replicate a protocell, say $\:m$. We will not use this parameter $\:m$ in any of the calculations. It also seems interesting to consider a nonlinear scenario where some $\:n$ molecules of food are ***simultaneously*** needed to create a protocell:

2 $$\:n\:X+U\to\:U+U{\prime\:}\:$$

We have run a few simulations using this equation (and with relevant changes to all other equations) and the only noticeable changes were to the first stage of evolution of the system. Larger than one values of $\:n$ resulted in faster evolution time of that stage. We will talk about that phenomenon in more detail below. Subsequently, we have used $\:n=1$ for all further calculations due to increased numerical stability and smaller calculation time in comparison to larger values of $\:n$.

We further consider that the protocells can “die off” without any mutual inhibition:

3 $$\:U\to\:W\:$$

where $\:W$ is some waste “molecule” and by waste “molecule” we mean that we also express waste in protocells, the same as food.

Finally, we need to close the model, and this is achieved by recycling the waste.

4 $$\:W\to\:X\:$$

The alternative is to introduce a pass-through model, where the food flows in at some constant rate and the waste is discarded. It can be shown that these models are dynamically nearly equivalent up to some time-dependent transformations. However, pass-through models don’t have a well-defined integral of motion, which makes them harder to model and account for errors. In addition, as the total amount of matter in a pass-through model linearly increases over time that makes such models less stable than the models with recycling. In other words, a pass-through model is substantially more likely to blow up numerically if some coefficients of the model are not chosen correctly.

Given the equations above, the changes in protocell concentration $\:U$ can be written as:

5 $$\:\frac{dU\left(\eta\:,\zeta\:,t\right)}{dt}={\rho\:}_{X}\left(t\right)\underset{-1}{\overset{1}{\int\:}}\underset{0}{\overset{L}{\int\:}}K\left(\eta\:,\zeta\:,{\eta\:}^{{\prime\:}},{\zeta\:}^{{\prime\:}}\right)\:U\left({\eta\:}^{{\prime\:}},{\zeta\:}^{{\prime\:}},t\right)\:d{\eta\:}^{{\prime\:}}\:d{\zeta\:}^{{\prime\:}}-{\gamma\:}_{0}\:\gamma\:\left(\eta\:,\zeta\:\right)\:U\left(\eta\:,\zeta\:,t\right)$$

where $\:U\left(\eta\:,\zeta\:,t\right)$ is a concentration of protocells with total enantioselectivity $\:\eta\:$ and the amount of stored information $\:\zeta\:$ at time $\:t$, $\:{\rho\:}_{X}\left(t\right)$ is the concentration of food molecules at time $\:t$, $\:K\left(\eta\:,\zeta\:,{\eta\:}^{{\prime\:}},{\zeta\:}^{{\prime\:}}\right)\:$is the rate at which protocells $\:U\left({\eta\:}^{{\prime\:}},{\zeta\:}^{{\prime\:}},t\right)$ can produce protocells $\:U\left(\eta\:,\zeta\:,t\right)$, $\:{\gamma\:}_{0}$ is a normalization constant, so that $\:\gamma\:\left(0,\:0\right)\equiv\:1$, and $\:{\gamma\:}_{0}\:\gamma\:\left(\eta\:,\zeta\:\right)$ is the decay rate of protocells $\:U\left(\eta\:,\zeta\:,t\right)$. The range of enantioselectivity is naturally the interval $\:\left[-1,\:1\right]$ with the initial value located near $\:\eta\:=0$ (nearly racemic mixture). The range in the information space depends on how we define the amount of information passed from generation to generation and what we mean as that information. However, we can always change the variables so that the domain in $\:\zeta\:$ starts from 0 and we keep the upper boundary as some value $\:L$. We can further rescale $\:\zeta\:$ so that to make that $\:L$ any value we want, e.g., 1, but we find it more convenient to keep it without rescaling in case we’d want to compare some models with different values of $\:L$ in the future. Subsequently, that makes the initial state of protocells as some small narrow peak near $\:\left(0,\:0\right)$ in $\:\left(\eta\:,\zeta\:\right)$ space.

The changes in $\:X$ and $\:W$ can be written as:

6 $$\:\frac{d{\rho\:}_{X}\left(t\right)}{dt}=\left(-{\rho\:}_{X}\left(t\right)\underset{-1}{\overset{1}{\int\:}}\underset{0}{\overset{L}{\int\:}}\underset{-1}{\overset{1}{\int\:}}\underset{0}{\overset{L}{\int\:}}K\left(\eta\:,\zeta\:,{\eta\:}^{{\prime\:}},{\zeta\:}^{{\prime\:}}\right)\:U\left({\eta\:}^{{\prime\:}},{\zeta\:}^{{\prime\:}},t\right)\:d{\eta\:}^{{\prime\:}}\:d{\zeta\:}^{{\prime\:}}\:d\eta\:\:d\zeta\:+s\:{\rho\:}_{W}\left(t\right)\right)\:$$

where $\:{\rho\:}_{W}\left(t\right)$ is the concentration of waste “molecules” at time $\:t$ and

7 $$\:\frac{d{\rho\:}_{W}\left(t\right)}{dt}=-s\:{\rho\:}_{W}\left(t\right)+{\gamma\:}_{0}\underset{-1}{\overset{1}{\int\:}}\underset{0}{\overset{L}{\int\:}}\:\gamma\:\left(\eta\:,\zeta\:\right)\:U\left(\eta\:,\zeta\:,t\right)\:d\eta\:\:d\zeta\:\:$$

where $\:s$ is a recycling rate.

We can first look at the stationary point of these equations. Equation (5) leads to the following condition:

8 $$\:\:\underset{-1}{\overset{1}{\int\:}}\underset{0}{\overset{L}{\int\:}}\frac{K\left(\eta\:,\zeta\:,{\eta\:}^{{\prime\:}},{\zeta\:}^{{\prime\:}}\right)\:}{\gamma\:\left(\eta\:,\zeta\:\right)}u\left({\eta\:}^{{\prime\:}},{\zeta\:}^{{\prime\:}}\right)\:d{\eta\:}^{{\prime\:}}\:d{\zeta\:}^{{\prime\:}}=\stackrel{\sim}{\lambda\:}\:u\left(\eta\:,\zeta\:\right)$$

where $\:\stackrel{\sim}{\lambda\:}=\frac{{\gamma\:}_{0}}{{\rho\:}_{X}}$, $\:U\left(\eta\:\right)\equiv\:{U}_{0}\:u\left(\eta\:\right)$ and $\:u\left(\eta\:,\zeta\:\right)$ is $\:{L}_{2}$ normalized so that:

9 $$\:\underset{-1}{\overset{1}{\int\:}}\underset{0}{\overset{L}{\int\:}}u{\left(\eta\:,\zeta\:\right)}^{2}\:d\eta\:\:d\zeta\:=1$$

This is a two-dimensional Fredholm integral operator of the first kind for the kernel $\:\stackrel{\sim}{K}\left(\eta\:,\zeta\:,{\eta\:}^{{\prime\:}},{\zeta\:}^{{\prime\:}}\right)=\frac{K\left(\eta\:,\zeta\:,{\eta\:}^{{\prime\:}},{\zeta\:}^{{\prime\:}}\right)}{\gamma\:\left(\eta\:,\zeta\:\right)}$. If $\:\eta\:$ and $\:\zeta\:$ subspaces are separable: $\:\stackrel{\sim}{K}\left(\eta\:,\zeta\:,{\eta\:}^{{\prime\:}},{\zeta\:}^{{\prime\:}}\right)\equiv\:{K}_{\eta\:}\left(\eta\:,{\eta\:}^{{\prime\:}}\right)\:{K}_{\zeta\:}\left(\zeta\:,{\zeta\:}^{{\prime\:}}\right)$ then we can express $\:u\left(\eta\:,\zeta\:\right)\equiv\:{u}_{\eta\:}\left(\eta\:\right)\:{u}_{\zeta\:}\left(\zeta\:\right)$ and then Eq. (8) also splits into one-dimensional equations.

### Kernel normalization

Before we proceed further, it is convenient to perform some transformations of the original kernel $\:K\left(\eta\:,\zeta\:,{\eta\:}^{{\prime\:}},{\zeta\:}^{{\prime\:}}\right)$. First, we can extract a normalization constant $\:{k}_{0}$:

10 $$\:\:{k}_{0}=\underset{-1}{\overset{1}{\int\:}}\underset{0}{\overset{L}{\int\:}}K\left(\eta\:,\zeta\:,0,\:0\right)\:d\eta\:\:d\zeta\:$$

which is a total production rate at a point $\:\left(0,\:0\right)$, and a total normalized replication rate $\:{k}_{a}\left({\eta\:}^{{\prime\:}},{\zeta\:}^{{\prime\:}}\right)$:

11 $$\:{k}_{a}\left({\eta\:}^{{\prime\:}},{\zeta\:}^{{\prime\:}}\right)=\frac{1}{{k}_{0}}\:\underset{-1}{\overset{1}{\int\:}}\underset{0}{\overset{L}{\int\:}}K\left(\eta\:,\zeta\:,{\eta\:}^{{\prime\:}},{\zeta\:}^{{\prime\:}}\right)\:d\eta\:\:d\zeta\:$$

Then we can rewrite the kernel as:

12 $$\:\:K\left(\eta\:,\zeta\:,{\eta\:}^{{\prime\:}},{\zeta\:}^{{\prime\:}}\right)\equiv\:{k}_{0}\:{k}_{a}\left({\eta\:}^{{\prime\:}},{\zeta\:}^{{\prime\:}}\right)\:p\left(\eta\:,\zeta\:,{\eta\:}^{{\prime\:}},{\zeta\:}^{{\prime\:}}\right)$$

where $\:p\left(\eta\:,{\eta\:}^{{\prime\:}},\zeta\:,{\zeta\:}^{{\prime\:}}\right)$ is defined as:

13 $$\:\:p\left(\eta\:,\zeta\:,{\eta\:}^{{\prime\:}},{\zeta\:}^{{\prime\:}}\right)\equiv\:\frac{K\left(\eta\:,\zeta\:,{\eta\:}^{{\prime\:}},{\zeta\:}^{{\prime\:}}\right)}{{k}_{0}\:{k}_{a}\left({\eta\:}^{{\prime\:}},{\zeta\:}^{{\prime\:}}\right)}$$

from which it follows that:

14 $$\:\underset{-1}{\overset{1}{\int\:}}\underset{0}{\overset{L}{\int\:}}\:p\left(\eta\:,\zeta\:,{\eta\:}^{{\prime\:}},{\zeta\:}^{{\prime\:}}\right)\:d\eta\:\:d\zeta\:=\underset{-1}{\overset{1}{\int\:}}\underset{0}{\overset{L}{\int\:}}\frac{K\left(\eta\:,\zeta\:,{\eta\:}^{{\prime\:}},{\zeta\:}^{{\prime\:}}\right)}{{k}_{0}\:{k}_{a}\left({\eta\:}^{{\prime\:}},{\zeta\:}^{{\prime\:}}\right)}\:d\eta\:\:d\zeta\:\equiv\:1$$

which means that $\:p\left(\eta\:,\zeta\:,{\eta\:}^{{\prime\:}},{\zeta\:}^{{\prime\:}}\right)$ is the probability that species with enantiomeric excess $\:{\eta\:}^{{\prime\:}}$ and amount of stored information $\:{\zeta\:}^{{\prime\:}}$ would produce species with total enantiomeric excess $\:\eta\:$ and amount of stored information $\:\zeta\:$. As mutations are small, that probability should be a narrow peak centered near the point $\:\left(\eta\:={\eta\:}^{{\prime\:}},\zeta\:={\zeta\:}^{{\prime\:}}\right)$.

Assuming that mutations in $\:\eta\:$ and $\:\zeta\:$ spaces are independent and taking into account that the number of molecules and protocells was very large, allows us to utilize Central Limit Theorem. That means that we can use normal distributions to model $\:p\left(\eta\:,{\eta\:}^{{\prime\:}},\zeta\:,{\zeta\:}^{{\prime\:}}\right)$ as some narrow peak near $\:\left({\eta\:}^{{\prime\:}},\:{\zeta\:}^{{\prime\:}}\right)$:

15 $$\:p\left(\eta\:,\zeta\:,{\eta\:}^{{\prime\:}},{\zeta\:}^{{\prime\:}}\right)=\frac{2\:{e}^{-\:\frac{{\left(\eta\:-{\eta\:}^{{\prime\:}}\right)}^{2}}{{\epsilon}_{\eta\:}^{2}}}}{{\epsilon}_{\eta\:}\:\sqrt{\pi\:}\:\left(\text{e}\text{r}\text{f}\left(\frac{1-{\eta\:}^{{\prime\:}}}{{\epsilon}_{\eta\:}}\right)+\text{e}\text{r}\text{f}\left(\frac{1+{\eta\:}^{{\prime\:}}}{{\epsilon}_{\eta\:}}\right)\right)}\:\bullet\:\:\frac{2\:{e}^{-\:\frac{{\left(\zeta\:-{\zeta\:}^{{\prime\:}}\right)}^{2}}{{\epsilon}_{\zeta\:}^{2}}}}{{\epsilon}_{\zeta\:}\:\sqrt{\pi\:}\:\left(\text{e}\text{r}\text{f}\left(\frac{L-{\zeta\:}^{{\prime\:}}}{{\epsilon}_{\zeta\:}}\right)+\text{e}\text{r}\text{f}\left(\frac{{\zeta\:}^{{\prime\:}}}{{\epsilon}_{\zeta\:}}\right)\right)}$$

where $\:{\epsilon}_{\eta\:}\ll\:1$, $\:{\epsilon}_{\zeta\:}\ll\:L$ are some small parameters (in general functions of $\:{\eta\:}^{{\prime\:}}$ and $\:{\zeta\:}^{{\prime\:}}$) defining mutation rates, $\:\text{e}\text{r}\text{f}$ is the error function, and the normalization coefficient follows from normalization condition Eq. (14). This probability is nearly identical to the two-dimensional normal probability distribution inside the domain: $\:{D}_{\epsilon}=\left[-1+6\:{\epsilon}_{\eta\:},\:1-6\:{\epsilon}_{\eta\:}\right]\times\:\left[6\:{\epsilon}_{\zeta\:},\:L-6\:{\epsilon}_{\eta\:}\right]$ and has some bias at the edges of the full domain $\:D=\left[-1,\:1\right]\times\:\left[0,\:L\right]$. This bias is irrelevant because the starting point $\:\left(\eta\:=0,\zeta\:=0\right)$ is in the middle of the domain in $\:\eta\:$ space and as we are interested in the system moving away from $\:\zeta\:=0$ some irregularities near that point at the beginning of the time evolution are insignificant.

After all these transformations, the Eq. (8) becomes:

16 $$\:\:\underset{-1}{\overset{1}{\int\:}}\underset{0}{\overset{L}{\int\:}}\frac{{k}_{a}\left({\eta\:}^{{\prime\:}},{\zeta\:}^{{\prime\:}}\right)\:p\left(\eta\:,\zeta\:,{\eta\:}^{{\prime\:}},{\zeta\:}^{{\prime\:}}\right)}{\gamma\:\left(\eta\:,\zeta\:\right)}\:u\left({\eta\:}^{{\prime\:}},{\zeta\:}^{{\prime\:}}\right)\:d{\eta\:}^{{\prime\:}}\:d{\zeta\:}^{{\prime\:}}=\lambda\:\:u\left(\eta\:,\zeta\:\right)$$

where $\:\lambda\:=\frac{{\gamma\:}_{0}}{{k}_{0}\:{\rho\:}_{X}}$. We can estimate the largest eigenvalue and approximate location of its eigenvector under reasonable assumptions that the mutations are small. Consider that the first eigenvector is a narrow “bump” function of some height $\:{u}_{0}$ with the widths (standard deviations) $\:{\sigma\:}_{\eta\:}$ and $\:{\sigma\:}_{\zeta\:}$ and it is centered near point $\:\left({\eta\:}_{0},\:{\zeta\:}_{0}\right).$ Then, we can integrate Eq. (16) by $\:d\zeta\:\:d\eta\:$ and approximate it. Then:

17 $$\:\underset{-1}{\overset{1}{\int\:}}\underset{0}{\overset{L}{\int\:}}u\left(\eta\:,\zeta\:\right)\:d\eta\:\:d\zeta\:\approx\:c\:{u}_{0}\:{\sigma\:}_{\eta\:}\:{\sigma\:}_{\zeta\:}$$

where $\:c$ is some numerical factor, which depends on the actual shape of $\:u\left(\eta\:,\zeta\:\right)$ and:

18 $$\begin{aligned}&\underset{-1}{\overset{1}{\int\:}}\underset{0}{\overset{L}{\int\:}}\:\underset{-1}{\overset{1}{\int\:}}\underset{0}{\overset{L}{\int\:}}\frac{{k}_{a}\left({\eta\:}^{{\prime\:}},{\zeta\:}^{{\prime\:}}\right)\:p\left(\eta\:,\zeta\:,{\eta\:}^{{\prime\:}},{\zeta\:}^{{\prime\:}}\right)}{\gamma\:\left(\eta\:,\zeta\:\right)}\:u\left({\eta\:}^{{\prime\:}},{\zeta\:}^{{\prime\:}}\right)\:d{\eta\:}^{{\prime\:}}\:d{\zeta\:}^{{\prime\:}}\:d\eta\:d\zeta \nonumber\\ &\quad \approx\:\underset{-1}{\overset{1}{\int\:}}\underset{0}{\overset{L}{\int\:}}\frac{{k}_{a}\left({\eta\:}^{{\prime\:}},{\zeta\:}^{{\prime\:}}\right)}{\gamma\:\left({\eta\:}^{{\prime\:}},{\zeta\:}^{{\prime\:}}\right)}\:\left(\underset{-1}{\overset{1}{\int\:}}\underset{0}{\overset{L}{\int\:}}p\left(\eta\:,\zeta\:,{\eta\:}^{{\prime\:}},{\zeta\:}^{{\prime\:}}\right)\:d\eta\:\:d\zeta\:\right)u\left({\eta\:}^{{\prime\:}},{\zeta\:}^{{\prime\:}}\right)\:d{\eta\:}^{{\prime\:}}\:d{\zeta\:}^{{\prime\:}}\nonumber\\ &\quad \approx\:\frac{{k}_{a}\left({\eta\:}_{0},{\zeta\:}_{0}\right)}{\gamma\:\left({\eta\:}_{0},{\zeta\:}_{0}\right)}\:c\:{u}_{0}\:{\sigma\:}_{\eta\:}\:{\sigma\:}_{\zeta\:}\end{aligned}$$

where the first approximate transition can be made because $\:p\left(\eta\:,\zeta\:,{\eta\:}^{{\prime\:}},{\zeta\:}^{{\prime\:}}\right)$ is non-zero when $\:\eta\:$ is close to $\:{\eta\:}^{{\prime\:}}$ and $\:\zeta\:$ is close to $\:{\zeta\:}^{{\prime\:}}$ (the mutations are considered small) and $\:\gamma\:\left(\eta\:,\zeta\:\right)$ is a slow function in the range where $\:p\left(\eta\:,\zeta\:,{\eta\:}^{{\prime\:}},{\zeta\:}^{{\prime\:}}\right)$ is non-zero (the decay rate for new protocells is not very different from the one of the original protocells), and the second approximation follows from the fact that we considered $\:u\left({\eta\:}^{{\prime\:}},{\zeta\:}^{{\prime\:}}\right)$ as a narrow peak near $\:\left({\eta\:}_{0},\:{\zeta\:}_{0}\right)$. This leads to an estimate:

19 $$\:\lambda\:\approx\:\frac{{k}_{a}\left({\eta\:}_{0},{\zeta\:}_{0}\right)}{\gamma\:\left({\eta\:}_{0},{\zeta\:}_{0}\right)}$$

And since we are interested in the largest eigenvalue, that means that the point $\:\left({\eta\:}_{0},{\zeta\:}_{0}\right)$ is where the function:

20 $$\:f\left(\eta\:,\zeta\:\right)=\frac{{k}_{a}\left(\eta\:,\zeta\:\right)}{\gamma\:\left(\eta\:,\zeta\:\right)}$$

has a global maximum on the domain: $\:\left[-1,\:1\right]\times\:\left[0,\:L\right]$.

### Diffusion

As the mutations are considered small, then $\:p\left(\eta\:,\zeta\:,{\eta\:}^{{\prime\:}},{\zeta\:}^{{\prime\:}}\right)$ is a narrow peak centered around the point $\:\left({\eta\:}^{{\prime\:}},{\zeta\:}^{{\prime\:}}\right)$. In this case we can perform a Taylor expansion of $\:U\left({\eta\:}^{{\prime\:}},{\zeta\:}^{{\prime\:}},t\right)$ near $\:U\left(\eta\:,\zeta\:,t\right)$ when $\:U$ is substantially wider than $\:p$:

21 $$\begin{aligned}&U\left({\eta\:}^{{\prime\:}},{\zeta\:}^{{\prime\:}},t\right)\approx\:U\left(\eta\:,\zeta\:,t\right)+\left({\eta\:}^{{\prime\:}}-\eta\:\right)\frac{\partial\:U\left(\eta\:,\zeta\:,t\right)}{\partial\:\eta\:}+\left({\zeta\:}^{{\prime\:}}-\zeta\:\right)\frac{\partial\:U\left(\eta\:,\zeta\:,t\right)}{\partial\:\zeta\:}\nonumber\\ &\quad +\frac{1}{2}\left({\left({\eta\:}^{{\prime\:}}-\eta\:\right)}^{2}\frac{{\partial\:}^{2}U\left(\eta\:,\zeta\:,t\right)}{\partial\:{\eta\:}^{2}}+2\left({\eta\:}^{{\prime\:}}-\eta\:\right)\left({\zeta\:}^{{\prime\:}}-\zeta\:\right)\frac{{\partial\:}^{2}U\left(\eta\:,\zeta\:,t\right)}{\partial\:\eta\:\:\partial\:\zeta\:}+{\left({\zeta\:}^{{\prime\:}}-\zeta\:\right)}^{2}\frac{{\partial\:}^{2}U\left(\eta\:,\zeta\:,t\right)}{\partial\:{\zeta\:}^{2}}\right)\end{aligned}$$

and substituting into Eq. (5) we can obtain:

22 $$\:\frac{\partial\:U}{\partial\:\tau\:}=\left(\alpha\:-\gamma\:\right)\:U+{\beta\:}_{\eta\:}\frac{\partial\:U}{\partial\:\eta\:}+{\beta\:}_{\zeta\:}\frac{\partial\:U}{\partial\:\zeta\:}+\frac{1}{2}\left({\delta\:}_{\eta\:\eta\:}\frac{{\partial\:}^{2}U}{\partial\:{\eta\:}^{2}}+2{\delta\:}_{\eta\:\zeta\:}\frac{{\partial\:}^{2}U}{\partial\:\eta\:\:\partial\:\zeta\:}+{\delta\:}_{\zeta\:\zeta\:}\frac{{\partial\:}^{2}U}{\partial\:{\zeta\:}^{2}}\right)$$

where:

23 $$\:\begin{array}{c}\tau\:={\gamma\:}_{0}\:t,\:U\equiv\:U\left(\eta\:,\zeta\:,t\right),\:r\equiv\:\frac{{k}_{0}}{{\gamma\:}_{0}}{\rho\:}_{X}\left(t\right)\\\:\:\alpha\:\equiv\:\alpha\:\left(\eta\:,\zeta\:,t\right)=r\underset{-1}{\overset{1}{\int\:}}\underset{0}{\overset{L}{\int\:}}{k}_{a}\left({\eta\:}^{{\prime\:}},{\zeta\:}^{{\prime\:}}\right)\:p\left(\eta\:,\zeta\:,{\eta\:}^{{\prime\:}},{\zeta\:}^{{\prime\:}}\right)\:d{\eta\:}^{{\prime\:}}\:d{\zeta\:}^{{\prime\:}},\\\:{\beta\:}_{\eta\:}\equiv\:{\beta\:}_{\eta\:}\left(\eta\:,\zeta\:,t\right)=r\underset{-1}{\overset{1}{\int\:}}\underset{0}{\overset{L}{\int\:}}\left({\eta\:}^{{\prime\:}}-\eta\:\right)\:{k}_{a}\left({\eta\:}^{{\prime\:}},{\zeta\:}^{{\prime\:}}\right)\:p\left(\eta\:,\zeta\:,{\eta\:}^{{\prime\:}},{\zeta\:}^{{\prime\:}}\right)\:d{\eta\:}^{{\prime\:}}\:d{\zeta\:}^{{\prime\:}}\\\:{\beta\:}_{\zeta\:}\equiv\:{\beta\:}_{\zeta\:}\left(\eta\:,\zeta\:,t\right)=r\underset{-1}{\overset{1}{\int\:}}\underset{0}{\overset{L}{\int\:}}\left({\zeta\:}^{{\prime\:}}-\zeta\:\right)\:{k}_{a}\left({\eta\:}^{{\prime\:}},{\zeta\:}^{{\prime\:}}\right)\:p\left(\eta\:,\zeta\:,{\eta\:}^{{\prime\:}},{\zeta\:}^{{\prime\:}}\right)\:d{\eta\:}^{{\prime\:}}\:d{\zeta\:}^{{\prime\:}}\\\:{\delta\:}_{\eta\:\eta\:}\equiv\:{\delta\:}_{\eta\:\eta\:}\left(\eta\:,\zeta\:,t\right)=r\underset{-1}{\overset{1}{\int\:}}\underset{0}{\overset{L}{\int\:}}{\left({\eta\:}^{{\prime\:}}-\eta\:\right)}^{2}\:{k}_{a}\left({\eta\:}^{{\prime\:}},{\zeta\:}^{{\prime\:}}\right)\:p\left(\eta\:,\zeta\:,{\eta\:}^{{\prime\:}},{\zeta\:}^{{\prime\:}}\right)\:d{\eta\:}^{{\prime\:}}\:d{\zeta\:}^{{\prime\:}}\\\:{\delta\:}_{\eta\:\zeta\:}\equiv\:{\delta\:}_{\eta\:\zeta\:}\left(\eta\:,\zeta\:,t\right)=r\underset{-1}{\overset{1}{\int\:}}\underset{0}{\overset{L}{\int\:}}\left({\eta\:}^{{\prime\:}}-\eta\:\right)\:\left({\zeta\:}^{{\prime\:}}-\zeta\:\right)\:{k}_{a}\left({\eta\:}^{{\prime\:}},{\zeta\:}^{{\prime\:}}\right)\:p\left(\eta\:,\zeta\:,{\eta\:}^{{\prime\:}},{\zeta\:}^{{\prime\:}}\right)\:d{\eta\:}^{{\prime\:}}\:d{\zeta\:}^{{\prime\:}}\\\:{\delta\:}_{\zeta\:\zeta\:}\equiv\:{\delta\:}_{\zeta\:\zeta\:}\left(\eta\:,\zeta\:,t\right)=r\underset{-1}{\overset{1}{\int\:}}\underset{0}{\overset{L}{\int\:}}{\left({\zeta\:}^{{\prime\:}}-\zeta\:\right)}^{2}\:{k}_{a}\left({\eta\:}^{{\prime\:}},{\zeta\:}^{{\prime\:}}\right)\:p\left(\eta\:,\zeta\:,{\eta\:}^{{\prime\:}},{\zeta\:}^{{\prime\:}}\right)\:d{\eta\:}^{{\prime\:}}\:d{\zeta\:}^{{\prime\:}}\end{array}\:\:$$

This means that Eq. (5) can be interpreted as time dependent convection-diffusion equation with a sink in the scenarios when the mutations are small and shape of $\:U$ is substantially wider than the width of mutation probability, though the form is slightly different from what is usually considered as convection-diffusion equation. It is important to note that both conditions: small mutation rate and abundance of species (which is a manifestation of the fact that $\:U$ is substantially wider than mutation probability) seem to hold in life and in the considered model for all values of time except some small amount of time at the beginning of the evolution of the system. The term $\:\left(\alpha\:-\gamma\:\right)$ is responsible for creation $\:\left(\alpha\:-\gamma\:\right)>0$ or destruction $\:\left(\alpha\:-\gamma\:\right)<0$ of protocells, vector $\:\beta\:=\left({\beta\:}_{\eta\:},\:{\beta\:}_{\zeta\:}\right)$ is the “speed” at which the species drift away, and matrix $$\:\delta\:=\left(\begin{array}{cc}{\delta\:}_{\eta\:\eta\:}&\:{\delta\:}_{\eta\:\zeta\:}\\\:{\delta\:}_{\zeta\:\eta\:}&\:{\delta\:}_{\zeta\:\zeta\:}\end{array}\right)$$ determines the “diffusion”. The drift is the most interesting factor here. We can perform a Taylor expansion of $\:{k}_{a}\left({\eta\:}^{{\prime\:}},{\zeta\:}^{{\prime\:}}\right)$ near the point $\:\left(\eta\:,\zeta\:\right)$:

24 $$\:{k}_{a}\left({\eta\:}^{{\prime\:}},{\zeta\:}^{{\prime\:}}\right)\approx\:{k}_{a}\left(\eta\:,\zeta\:\right)+\left({\eta\:}^{{\prime\:}}-\eta\:\right)\frac{\partial\:{k}_{a}\left(\eta\:,\zeta\:\right)}{\partial\:\eta\:}+\left({\zeta\:}^{{\prime\:}}-\zeta\:\right)\frac{\partial\:{k}_{a}\left(\eta\:,\zeta\:\right)}{\partial\:\zeta\:}$$

and then consider that $\:p\left(\eta\:,\zeta\:,{\eta\:}^{{\prime\:}},{\zeta\:}^{{\prime\:}}\right)$ is a nearly symmetric function near that point $\:\left({\eta\:}^{{\prime\:}},{\zeta\:}^{{\prime\:}}\right)$ inside $\:{D}_{\epsilon}$. Then:

25 $$\:\begin{array}{c}\:\alpha\:\approx\:r\:{k}_{a}\left(\eta\:,\zeta\:\right)\:,\\\:{\beta\:}_{\eta\:}\approx\:\frac{1}{2}\:r\:\frac{\partial\:{k}_{a}\left(\eta\:,\zeta\:\right)}{\partial\:\eta\:}{\epsilon\:}_{\eta\:}^{2}\:,\:{\beta\:}_{\zeta\:}\approx\:\frac{1}{2}\:r\:\frac{\partial\:{k}_{a}\left(\eta\:,\zeta\:\right)}{\partial\:\zeta\:}{\epsilon\:}_{\zeta\:}^{2}\:,\\\:{\delta\:}_{\eta\:\eta\:}\approx\:\frac{1}{2}\:r\:{k}_{a}\left(\eta\:,\zeta\:\right){\epsilon\:}_{\eta\:}^{2}\:,\:{\delta\:}_{\eta\:\zeta\:}\approx\:0,\:{\delta\:}_{\zeta\:\zeta\:}\approx\:\frac{1}{2}\:r\:{k}_{a}\left(\eta\:,\zeta\:\right){\epsilon\:}_{\zeta\:}^{2}\end{array}$$

and where:

26 $$\:\begin{array}{c}\:\underset{-1}{\overset{1}{\int\:}}\underset{0}{\overset{L}{\int\:}}{\left({\eta\:}^{{\prime\:}}-\eta\:\right)}^{2}\:p\left(\eta\:,\zeta\:,{\eta\:}^{{\prime\:}},{\zeta\:}^{{\prime\:}}\right)\:d{\eta\:}^{{\prime\:}}\:d{\zeta\:}^{{\prime\:}}\equiv\:\frac{{\epsilon\:}_{\eta\:}^{2}}{2},\\\:\underset{-1}{\overset{1}{\int\:}}\underset{0}{\overset{L}{\int\:}}{\left({\zeta\:}^{{\prime\:}}-\zeta\:\right)}^{2}\:p\left(\eta\:,\zeta\:,{\eta\:}^{{\prime\:}},{\zeta\:}^{{\prime\:}}\right)\:d{\eta\:}^{{\prime\:}}\:d{\zeta\:}^{{\prime\:}}\equiv\:\frac{{\epsilon\:}_{\zeta\:}^{2}}{2}\end{array}$$

and we ignored higher order terms by $\:{\epsilon}_{\eta\:}$ and $\:{\epsilon}_{\zeta\:}$. That means that the drift is determined by a vector $\:\overrightarrow{\beta\:}\:\sim\:\left(\frac{\partial\:{k}_{a}\left(\eta\:,\zeta\:\right)}{\partial\:\eta\:},\:\frac{\partial\:{k}_{a}\left(\eta\:,\zeta\:\right)}{\partial\:\zeta\:}\right)$, which is a local gradient of $\:{k}_{a}\left(\eta\:,\zeta\:\right)$ up to some coefficient of proportionality. We also note that $\:{\epsilon\:}_{\eta\:}={\epsilon}_{\eta\:}$ and $\:{\epsilon\:}_{\zeta\:}={\epsilon}_{\zeta\:}$ with a very high precision within $\:{D}_{\epsilon}$.

### Stages of evolution with time

We note that $\:{k}_{a}\left(\eta\:,\zeta\:\right)$ must be an even function of $\:\eta\:$ due to $\:L\leftrightarrow\:D$ symmetry and therefore all odd derivatives of $\:{k}_{a}\left(\eta\:,\zeta\:\right)$ by $\:\eta\:$ at the point $\:\left(0,\:0\right)$ must be zeros. Therefore, second order Taylor expansion of $\:{k}_{a}$ near point $\:\left(0,\:0\right)$ should not have terms linear in $\:\eta\:$:

27 $$\:{k}_{a}\left(\eta\:,\zeta\:\right)=1+\zeta\:\:\frac{\partial\:{k}_{a}\left(\eta\:,\zeta\:\right)}{\partial\:\zeta\:}+\frac{1}{2}\left({\eta\:}^{2}\:\frac{{\partial\:}^{2}{k}_{a}\left(\eta\:,\zeta\:\right)}{\partial\:{\eta\:}^{2}}+{\zeta\:}^{2}\:\frac{{\partial\:}^{2}{k}_{a}\left(\eta\:,\zeta\:\right)}{\partial\:{\zeta\:}^{2}}\right)$$

or, if we want to keep $\:{k}_{a}\left(\eta\:,\zeta\:\right)$ in a separable form then:

28 $$\:{k}_{a}\left(\eta\:,\zeta\:\right)\equiv\:{k}_{\eta\:}\left(\eta\:\right)\:{k}_{\zeta\:}\left(\zeta\:\right)=\left(1+\frac{{\eta\:}^{2}}{2}\:\frac{{\partial\:}^{2}{k}_{a}\left(\eta\:,\zeta\:\right)}{\partial\:{\eta\:}^{2}}\right)\left(1+\zeta\:\:\frac{\partial\:{k}_{a}\left(\eta\:,\zeta\:\right)}{\partial\:\zeta\:}+\frac{{\zeta\:}^{2}}{2}\:\frac{{\partial\:}^{2}{k}_{a}\left(\eta\:,\zeta\:\right)}{\partial\:{\zeta\:}^{2}}\right)$$

The latter form has some extra higher than level 2 terms in comparison to the Taylor expansion. This only affects the evolution in diagonal directions and only when cross terms become substantial. If both quadratic terms are positive (which is the case in life, except probably near some catastrophic events, which we are not considering here), then value of $\:{k}_{a}$ in separable form is larger in diagonal directions than when using the Taylor expansion. However, we have not seen the system going in diagonal directions even though we used a separable version of $\:{k}_{a}$ in most of our calculations.

Assuming that the mutations are small, we can consider various stages of evolution of the system. The initial state of the system is a very narrow peak near point $\:\left(0,\:0\right)$ in $\:\left(\eta\:,\zeta\:\right)$ space. If we replace $\:U\left(\eta\:,\zeta\:,t\right)={U}_{0}\left(t\right)\:\delta\:\left(\eta\:,\zeta\:\right)$ where $\:\delta\:\left(\eta\:,\zeta\:\right)$ is the Dirac delta function, then we can perform integrations in Eqs. (5–7) to obtain:

29 $$\:\frac{d{U}_{0}\left(t\right)}{dt}=\left(\:{k}_{0}\:{\rho\:}_{X}\left(t\right)-{\gamma\:}_{0}\right)\:{U}_{0}\left(t\right)$$

30 $$\:\frac{d{\rho\:}_{X}\left(t\right)}{dt}=n\:\left(-{k}_{0}\:{\rho\:}_{X}\left(t\right)\:{U}_{0}\left(t\right)+s\:{\rho\:}_{W}\left(t\right)\right)\:$$

31 $$\:\frac{d{\rho\:}_{W}\left(t\right)}{dt}=-s\:{\rho\:}_{W}\left(t\right)+{\gamma\:}_{0}\:{U}_{0}\left(t\right)\:$$

Provided that initially the food is abundant, and the initial number of protocells is very small, the first stage of evolution is an exponential growth of the total number of protocells with the initial growth rate: $\:\left(\:{k}_{0}\:{\rho\:}_{X}\left(0\right)-{\gamma\:}_{0}\right)$. The time interval of that period is when the initial number of protocells: $\:{U}_{0}\left(0\right)$ would exponentially grow to the order of $\:{\rho\:}_{X}\left(0\right)$:

32 $$\:{U}_{0}\left(0\right)\:{e}^{\left(\:{k}_{0}\:{\rho\:}_{X}\left(0\right)-{\gamma\:}_{0}\right)\:{t}_{0}}\sim{\rho\:}_{X}\left(0\right)$$

from which it follows that:

33 $$\:{t}_{0}\:\sim\:\frac{ln\left(\frac{{\rho\:}_{X}\left(0\right)}{{U}_{0}\left(0\right)}\right)}{{k}_{0}\:{\rho\:}_{X}\left(0\right)-{\gamma\:}_{0}}$$

When the food is abundant (as it is in the initial stage), then $\:{k}_{0}\:{\rho\:}_{X}\left(0\right)\gg\:{\gamma\:}_{0}$ and so we can ignore $\:{\gamma\:}_{0}$ in the denominator. Taking just one initial protocell and the total number of organic molecules or even all atoms on Earth as the hard limit gives an estimate that the logarithm in numerator is naturally limited to somewhere between ${10^1} \div {10^2}$. The actual value is irrelevant as the process is very quick. The value $\:{k}_{0}\:{\rho\:}_{X}\left(0\right)$ is essentially a doubling rate (to be more precise the time to increase the population in $\:e\approx\:2.7$ times) and so using even the upper (unrealistic) boundary of the nominator as $\:{10}^{2}$ and unrealistically slow doubling rate of protocells of, say, one year gives $\:{t}_{0}<{10}^{2}$ years, which is negligible on a geological time scale. This time period, $\:{t}_{0}$ is the amount of time, which would take the most inefficient protocells (located near point $\:\left(0,\:0\right)$ in $\:\left(\eta\:,\zeta\:\right)$ space) to consume nearly all food. If we look at Eq. (22), then $\:{t}_{0}$ can be interpreted as the period of time during which the value $\:\left(\alpha\:-\gamma\:\right)$ reaches 0 at point $\:\left(0,\:0\right)$:

34 $$\:\alpha\:\left(0,\:0,\:{t}_{0}\right)-\gamma\:\left(0,\:0\right)=\frac{{k}_{0}}{{\gamma\:}_{0}}{\rho\:}_{X}\left({t}_{0}\right){k}_{a}\left(0,\:0\right)-\gamma\:\left(0,\:0\right)=\frac{{k}_{0}}{{\gamma\:}_{0}}{\rho\:}_{X}\left({t}_{0}\right)-1=0$$

Once this point in time is reached, then further evolution of the system is only possible by increasing the efficiency of protocells. The less efficient protocells then will die off whereas the more efficient protocells will continue to evolve thus making it possible to sustain their replication at smaller and smaller concentrations of food. That means that the boundary:

35 $$\:\alpha\:\left(\eta\:,\zeta\:,\:t\right)-\gamma\:\left(\eta\:,\zeta\:\right)=0$$

should move away from point $\:\left(0,\:0\right)$ as the system evolves.

The second stage of evolution substantially depends on the values of the drift vector, $\:\overrightarrow{\beta\:}$. The value $\:{\beta\:}_{\eta\:}\left(\text{0,0}\right)\equiv\:0$ due to symmetry, however $\:{\beta\:}_{\zeta\:}\left(\text{0,0}\right)$ does not have to be zero. If it is zero or close to zero, then the second stage of evolution is a diffusion in $\:\left(\eta\:,\zeta\:\right)$ space until the time when quadratic coefficients in $\:{k}_{a}$ start to play a role. However, if $\:{\beta\:}_{\zeta\:}\left(\text{0,0}\right)$ is large enough, then the initial bump near $\:\left(\text{0,0}\right)$ will move in $\:\zeta\:$ space faster than it diffuses. Biologically this means that in the first case the system produces a variety of species that are diverse both in total enantiomeric excess and the amount of stored information, whereas in the second case the species form a compact “bump” of not yet separated into left or right species, which quickly advance in the ability to pass information from generation to generation.

The third stage of evolution starts when quadratic coefficients in Eq. (22) become substantial. There is an extensive discussion in^23^ regarding how replication with errors helps to transition replicated structures toward homochirality. And while it is intuitively clear that passing more information from generation to generation is beneficial for the evolution of life, we will not dwell in theoretical considerations of this matter in the current work. Rather, we note, that if any of the quadratic coefficients were negative, then the diffusion in that direction would have been suppressed and subsequently life, as we know it, would not exist. Therefore, both quadratic coefficients must be positive. The time evolution in that stage is complicated enough so that it is not possible to deduce how the system should evolve without actually solving the dynamical problem. Before we get to that, we shall note the following. The drift vector, $\:\overrightarrow{\beta\:}$ is proportional to the gradient of $\:{k}_{a}$ at a given point. Therefore, the further is the point from $\:\left(\text{0,0}\right)$ in any direction, the larger is that gradient and therefore the faster the protocells that are at that point move in the direction of that gradient because $\:{k}_{a}$ has positive quadratic coefficients in the Taylor expansion in both directions. If we note that the evolution equation resembles the convection-diffusion equation, then we can talk about the speed at which the width of the initial spike increases. And it is a well-known fact that a standard diffusion equation with constant diffusion coefficient has a Gaussian solution with the width $\:\sim\sqrt{D\:t}\:$where $\:D$ is the diffusion coefficient and $\:t$ is evolution time. Therefore the “speed” at which the edge of the bump moves is $\:\sim\sqrt{\frac{D}{t}}\:$ (as a derivative of the width by time). This can be estimated in the region where quadratic coefficients give substantial contribution to $\:{k}_{a}$ (for example for large enough values of $\:\zeta\:$) as $\:\sim\frac{\zeta\:}{\sqrt{t}}$ whereas $\:{\beta\:}_{\zeta\:}\:\sim\:\zeta\:$. Another critical fact comes from stochasticity related to chemical evolution. A stochasticity in relation to the current model means that there is a probability that several favorable mutations happen in a row, and which would place some protocells in the region with higher amplification and subsequently drift speed. When that happens and because the speed of the edge of the bump decreases with time, then it could be possible for the protocells with favorable mutations to get separated from the rest of the pack. That’s the scenario that we are the most interested in when solving dynamical evolution problem.

## Solution of dynamical problems

The continuous representation is well-suitable for finding the stationary points of the considered system and performing some simplified analysis. However, it breaks down when we want to consider a dynamical evolution, that is, how does the system get to that stationary point from the initial state? This is mostly due to machine noise. As the number of calculations on each step is very large, the machine noise produces some very small yet non-zero values of $\:U\left(\eta\:,\zeta\:,t\right)$ far from the initial state near $\:\left(0,\:0\right)$. That machine noise is also likely to appear in the regions of $\:{k}_{a}$ with large values and subsequently with larger “amplification”. As a result, the machine noise will experience exponential growth and that results in some artefacts appearing where they are not supposed to be.

As chemical systems evolve in integer numbers rather than in real numbers, we could try using some version of chemical master equation to calculate the evolution of the system in the number of molecules. Then very small real numbers cannot appear in the regions where they are not supposed to be and so the issue of unrealistic artefacts is removed. Since we want to use a large number of molecules and protocells in our calculations, Gillespie tau-leaping algorithm^28^ is well suitable for such a problem. We have used a custom version of this algorithm to ensure stability near zero values. It is interesting to note that the Gillespie tau-leaping algorithm is essentially a stochastic version of a simple Euler algorithm for solving differential equations. In fact, it is straightforward to show that as the number of molecules goes to infinity, the Gillespie tau-leaping algorithm converges to the Euler algorithm. This means that the Gillespie tau-leaping algorithm should be plagued by the same issues as the Euler algorithm. In particular, it means that the Gillespie tau-leaping algorithm should undershoot for exponential growth and overshoot for exponential decay, of which the exponential decay is the most dangerous. The algorithm can easily overshoot below zero, after which all the calculations break down. This is why chemical equations are considered stiff. When solving differential equations this is resolved either by substantially reducing the time step, thus drastically increasing the solution time, or using higher-order direct methods, or using indirect methods, which require inverting matrices. Apart from the need to adapt indirect methods for the Gillespie tau-leaping algorithm, this is simply not suitable for the current problem because, for example, a 500 × 500 grid has 250,000 variables and using indirect method would require inverting 250,000 × 250,000 matrices, where machine noise, calculation time, and memory requirements would make it prohibitive. Using higher order direct methods with the tau-leaping algorithm would also require adaptation.

The solution to this problem in case of chemical equations is simple. We let the system overshoot into negative values but treat all negative values as exact zeros in subsequent calculations. This is the approach that we used in some previous works, and it showed results consistent with the ones obtained using more advanced but slower algorithms. Out of all the variables in the system considered in the current work it is food that could easily become negative at some steps, especially if some of the parameters are large enough. Even though the approach described above works even in such extreme cases, we fine-tune the algorithm further by adjusting the step so that to consume all food but no more than is available on a given step. The solution is stable and since the system has an invariant of motion, we can monitor that it is conserved exactly at each step.

## Results

We used the following separable approximations of $\:{k}_{a}\left(\eta\:,\zeta\:\right)\equiv\:{k}_{\eta\:}\left(\eta\:\right)\:{k}_{\zeta\:}\left(\zeta\:\right)$ and $\:\gamma\:$ in the current work:

36 $$\:x=c\:\zeta\:$$

37 $$\:{k}_{a}\left(\eta\:,\zeta\:\right)\equiv\:{k}_{\eta\:}\left(\eta\:\right)\:{k}_{\zeta\:}\left(\zeta\:\right)=\left(1+\frac{{b}_{\eta\:}\:{\eta\:}^{2}}{2}\right)\left(1+{a}_{\zeta\:}\:x+\frac{{b}_{\zeta\:}\:{x}^{2}}{2}\right)$$

38 $$\:\gamma\:\left(\eta\:,\zeta\:\right)=\left(1-g\:\eta\:\right)\left(1+\frac{f\:{x}^{8}}{8!}\right)$$

Parameter $\:g$ is a small linear global asymmetry factor and our view is that it should be introduced in the decay rate $\:\gamma\:$ rather than in replication rate $\:{k}_{a}$ because the global asymmetry factor influences the stability of the left vs. right amino acids and sugars, which subsequently affects the lifespan of protocells. Parameter $\:f$ is an artificially introduced limiting factor to keep the stationary point off the boundary $\:\zeta\:=L$. If this factor is not introduced, then the stationary point in $\:\zeta\:$ space becomes near point $\:\zeta\:=L$. Another view is that any model should have limitations, and we wanted to limit how far our model could evolve. Parameter $\:c$ is a scaling coefficient, which we introduced for convenience.

One of the most important parameters in the Gillespie tau-leaping algorithm is the step size when all other parameters are fixed. However, we can always rescale the parameters so that to set step size to some desired value. We used fixed step size $\:\varDelta\:t=1$ and then set other parameters as necessary. This allows us to treat $\:\varDelta\:t$ as evolution epochs and it makes it easier to interpret the results, e.g., if $\:\varDelta\:t=1$ day, then $\:{\gamma\:}_{0}=0.01$ means that average protocell lifespan is: $\:\frac{1}{{\gamma\:}_{0}}=100$ days.

We used the following parameter values in calculations:

**Table:** 

Parameter	Value	Description
$\:d$	500	Size of the grid used in calculations. We used a $\:d\times\:d$ grid.
$\:L$	25	Upper boundary of the domain in $\:\zeta\:$ space. Currently the choice is arbitrary as we can always rescale the $\:\zeta\:$ variable. However, if we consider it as the natural logarithm of bits of information passed from generation to generation, then that value translates into $\:\approx\:{10}^{10}$ bytes, which by far exceeds reasonable estimates of how much information the protocells could have passed from generation to generation.
$\:c$	$\:\frac{3}{2\:L}$	Scaling coefficient to make it more convenient to set values of parameters in $\:\zeta\:$ dimension. This choice of $\:c$ was determined by our desire to scale $\:\frac{2}{3\:}L$ into 1 in scaled coordinate $\:x$.
$\:{k}_{0}$	0.1	The parameter $\:{k}_{0}$, as determined by Eq. (10), can be used as is when using ODE evolution. The value of $\:\frac{{k}_{0}}{{\rho\:}_{0}^{n}}$ is used instead when using Gillespie tau-leaping evolution in integer numbers. This adjustment is necessary because the term with $\:K$ in the original differential Eq. (5) is $\:\sim{\rho\:}^{n+1}$ and therefore $\:{k}_{0}$ has a dimension $\:\sim{\rho\:}^{-n}$. Therefore, we need to account for that when transitioning to the equations expressed in the number of molecules.
$\:{\gamma\:}_{0}$	0.01	Decay rate of protocells at point $\:\left(0,\:0\right)$.
$\:s$	1	Recycling rate. The value of that parameter determines how much residual waste is near the stationary point.
$\:{b}_{\eta\:}$	1	The value of $\:\frac{{\partial\:}^{2}{k}_{a}\left(\eta\:,\zeta\:\right)}{\partial\:{\eta\:}^{2}}$ at point $\:\left(0,\:0\right)$.
$\:{a}_{\zeta\:}$	$\:0$ or $\:1$	The value of $\:\frac{\partial\:{k}_{a}\left(\eta\:,\zeta\:\right)}{\partial\:\zeta\:}$ at point $\:\left(0,\:0\right)$.
$\:{b}_{\zeta\:}$	1	The value of $\:\frac{{\partial\:}^{2}{k}_{a}\left(\eta\:,\zeta\:\right)}{\partial\:{\zeta\:}^{2}}$ at point $\:\left(0,\:0\right)$.
$\:{\epsilon}_{\eta\:}$	0.005 or 0.01	Mutation rate in $\:\eta\:$ space.
$\:{\epsilon}_{\zeta\:}$	0.005 or 0.01	Mutation rate in $\:\zeta\:$ space.
$\:z$	$\:{10}^{-5}$	Threshold parameter to control the values of probability Eq. (15) to treat as exact zero. When $\:p\left(\eta\:,\zeta\:,{\eta\:}^{{\prime\:}},{\zeta\:}^{{\prime\:}}\right)<z$ we treat it as exact zero and remove it from the kernel function (but adjust total probability to sum up to 1). This allows us to consider a four-dimensional kernel as a sparse array resulting in a very substantial speed increase of the algorithm. As typical number of such non-zero points in $\:p\left(\eta\:,\zeta\:,{\eta\:}^{{\prime\:}},{\zeta\:}^{{\prime\:}}\right)$ is somewhere between 10 to 100 instead of $\:d\times\:d=\text{250,000}$, which results in a 1,000- to 10,000-fold speed increase of the algorithm.
$\:g$	$\:0.002\:$or $\:0.005$	Global asymmetry factor. The values smaller than $\:0.002$ are beyond the resolution of the grid used in our calculations.
$\:f$	1000	This is an artificial parameter to keep the stationary point off the upper boundary in $\:\zeta\:$ space and model the scenario when further increase of stored information results in faster decay rate. If this parameter is not introduced, the system will run toward the $\:\zeta\:=L$ edge of the domain, which produces harder to visualize results.
$\:{\rho\:}_{0}$	$\:{10}^{9}\div {10}^{18}$	Total number of molecules in the system. This is an invariant, and this number does not change over the course of evolution of the system. The bigger is $\:{\rho\:}_{0}$ the more the dynamical evolution starts to resemble evolution of the system in real numbers.
$\:{U}_{00}$	$\:{10}^{3}$	Initial number of protocells located at point $\:\left(0,\:0\right)$ at a time $\:t=0$.

The following scenarios were chosen for this article: $\:{b}_{\eta\:}=1$, $\:{b}_{\zeta\:}=1$, $\:g=0.002$, $\:{\rho\:}_{0}={10}^{18}$, $\:{a}_{\zeta\:}=0$ or 1, $\:{\epsilon}_{\eta\:}={\epsilon}_{\zeta\:}=0.005$ or $\:0.01$ and we summarized them in the Table 1.

**Table 1:** Parameters of the models considered in the current work.

No	Model parameters	Model code
1 (a)	$\:{b}_{\eta\:}=1$, $\:{a}_{\zeta\:}=0$, $\:{b}_{\zeta\:}=1$, $\:{\epsilon}_{\eta\:}={\epsilon}_{\zeta\:}=0.005$, $\:g=0.002$, $\:{\rho\:}_{0}={10}^{18}$	d500k1e005g01a002f1E
2 (b)	$\:{b}_{\eta\:}=1$, $\:{a}_{\zeta\:}=0$, $\:{b}_{\zeta\:}=1$, $\:{\epsilon}_{\eta\:}={\epsilon}_{\zeta\:}=0.01$, $\:g=0.002$, $\:{\rho\:}_{0}={10}^{18}$	d500k1e01g01a002f1E
3 (c)	$\:{b}_{\eta\:}=1$, $\:{a}_{\zeta\:}=1$, $\:{b}_{\zeta\:}=1$, $\:{\epsilon}_{\eta\:}={\epsilon}_{\zeta\:}=0.005$, $\:g=0.002$, $\:{\rho\:}_{0}={10}^{18}$	d500k1e005g01a002i10f1E
4 (d)	$\:{b}_{\eta\:}=1$, $\:{a}_{\zeta\:}=1$, $\:{b}_{\zeta\:}=1$, $\:{\epsilon}_{\eta\:}={\epsilon}_{\zeta\:}=0.01$, $\:g=0.002$, $\:{\rho\:}_{0}={10}^{18}$	d500k1e01g01a002i10f1E

**Fig. 1:**
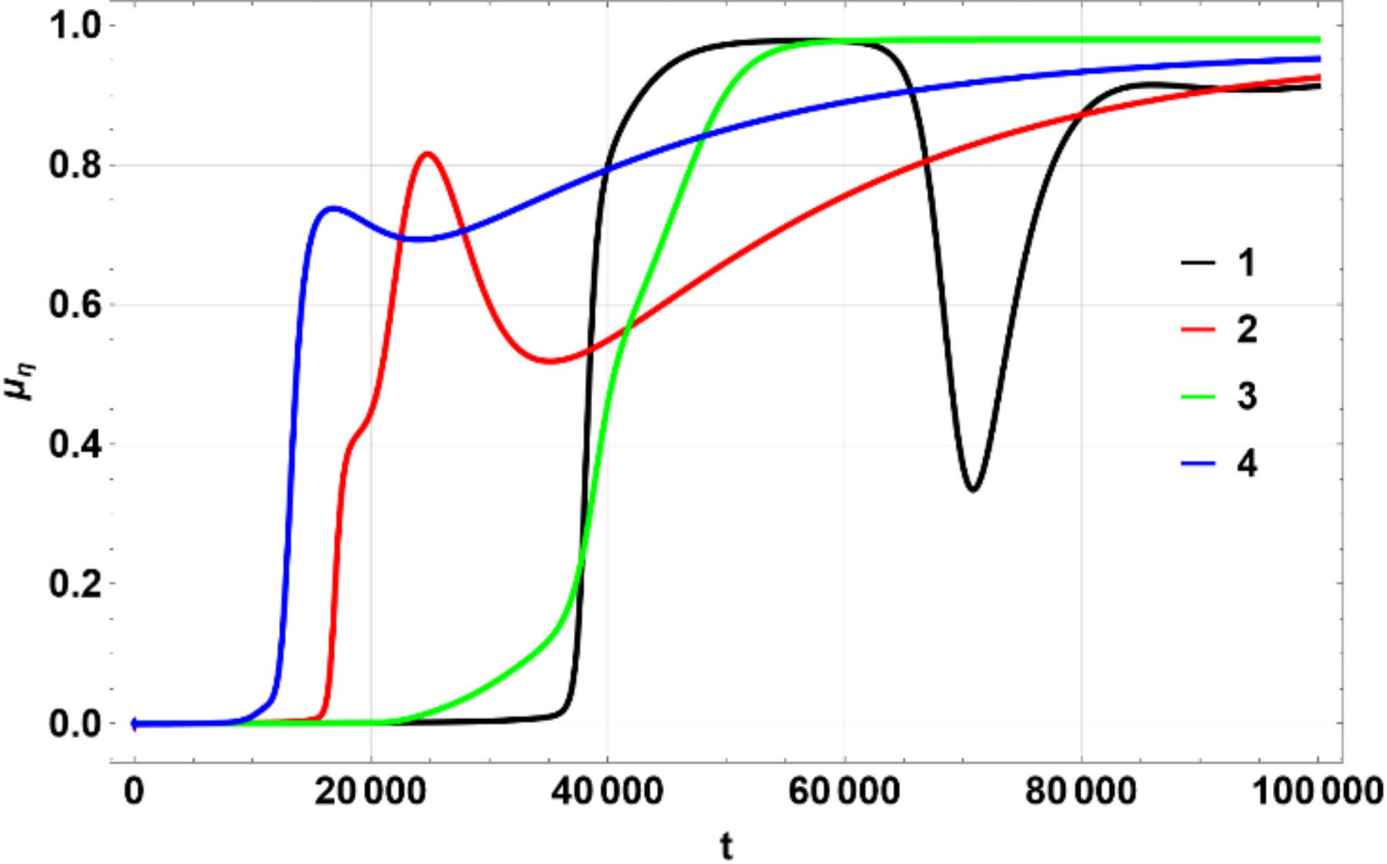
Dependence of $\:{\mu\:}_{\eta\:}$ on $\:t$. Produced using Wolfram Mathematica 13.

**Fig. 2:**
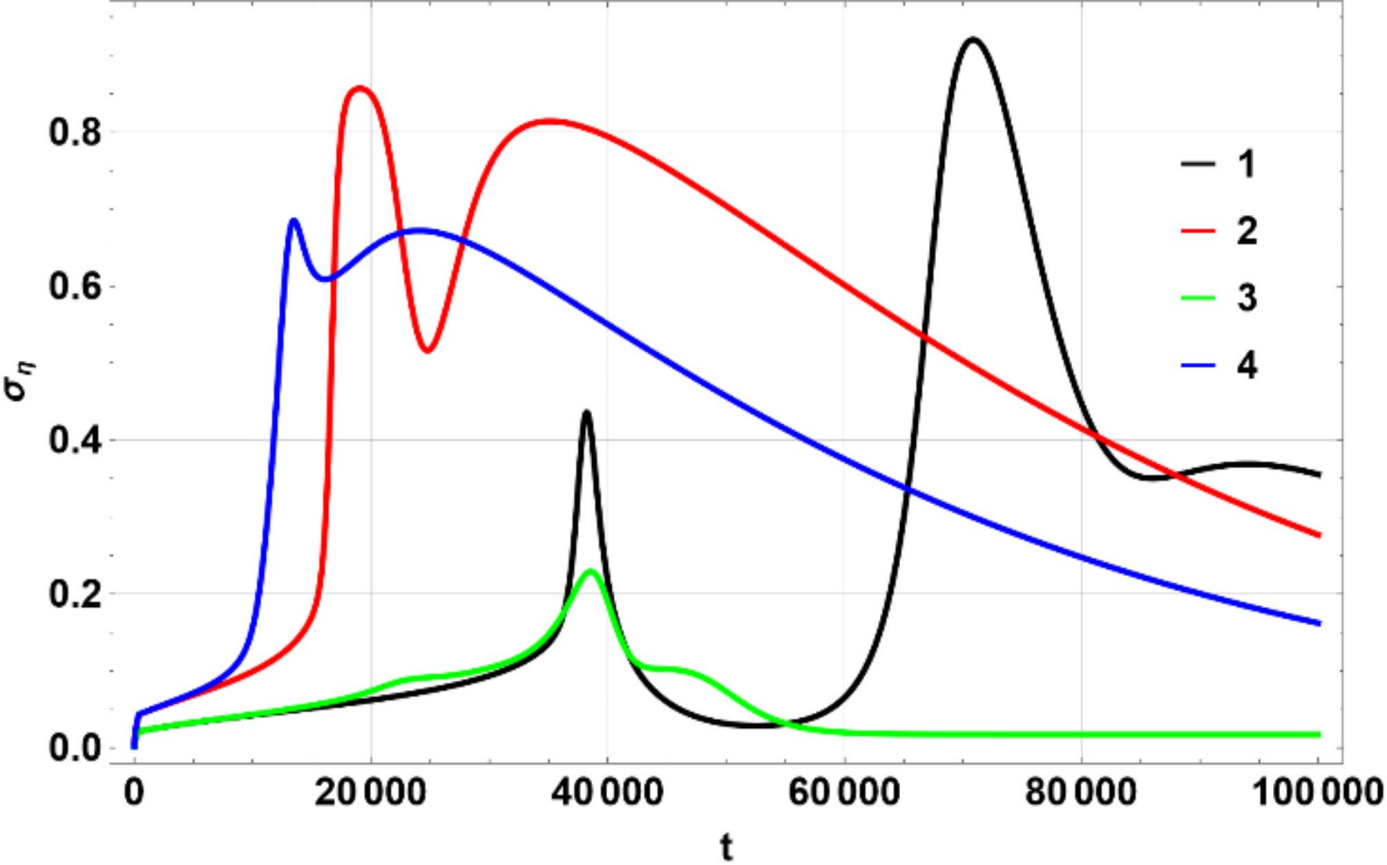
Dependence of $\:{\sigma\:}_{\eta\:}$ on $\:t$. Produced using Wolfram Mathematica 13.

The colors matching a number in the table above were used on multi-line 2D figures and 3D figures use (a) – (d) marking. 2D charts show means and widths (standard deviation) of $\:U\left(\eta\:,\zeta\:\right)$ over time of which the mean in $\:\eta\:$ space, $\:{\mu\:}_{\eta\:}$ (Fig. 1) and standard deviation $\:{\sigma\:}_{\eta\:}$, Fig. 2 are the most important. The 3D figures (distributions of $\:U\left(\eta\:,\zeta\:\right)$) are shown for some chosen values of time to illustrate the most interesting effects. All values of $\:U$ on 3D figures are in percent.

**Fig. 3:**
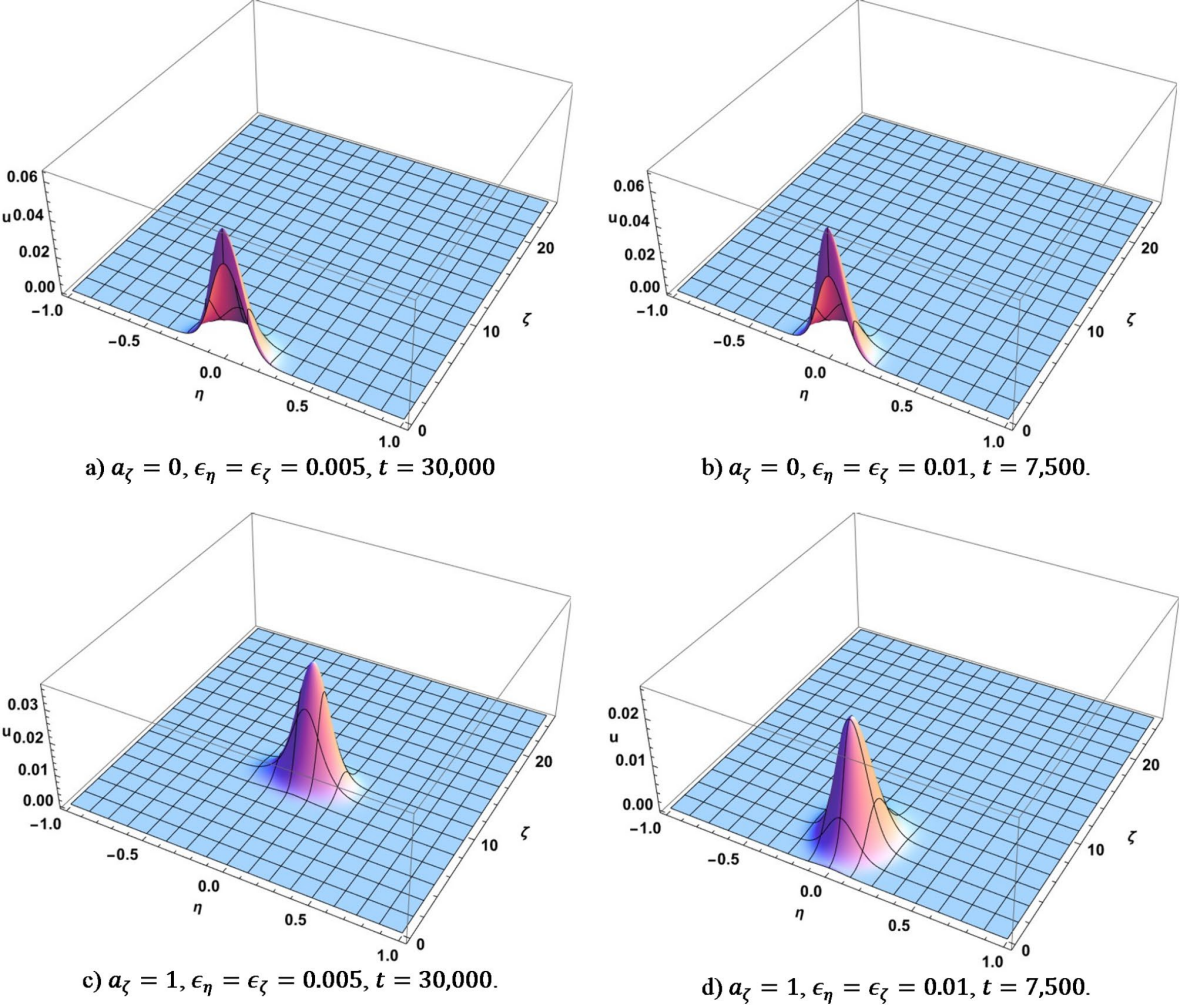
Dependence of $\:U$ on $\:\eta\:$ and $\:\zeta\:$ when the second stage of evolution (diffusion) has not completed yet. Produced using Wolfram Mathematica 13.

The first two stages of evolution (very quick exponential growth of the initial narrow bump followed by a diffusion-like spreading) were consistently observed among all runs that we performed. Figure 3 shows $\:U\left(\eta\:,\zeta\:\right)$ for some chosen values of time when the second stage of evolution has not completed yet. The time is $\:t=\text{30,000}$ for the case when $\:{\epsilon}_{\eta\:}={\epsilon}_{\zeta\:}=0.005$ and $\:t=\text{7,500}$ when $\:{\epsilon}_{\eta\:}={\epsilon}_{\zeta\:}=0.01$. The width of Gaussian solution of the diffusion equation with constant diffusion coefficient grows $\:\sim\sqrt{D\:t}\:\sim\:\epsilon\:\sqrt{\:t}$ ($\:\epsilon$ is either $\:{\epsilon}_{\eta\:}$ or $\:{\epsilon}_{\zeta\:}$). The widths of the bumps for $\:{\epsilon}_{\eta\:}={\epsilon}_{\zeta\:}=0.005$ vs. $\:{\epsilon}_{\eta\:}={\epsilon}_{\zeta\:}=0.01$ are nearly the same on the Figure 3 even though the evolution time is 4x faster, which is consistent with the estimate. There is a drift toward higher information content when $\:{a}_{\zeta\:}=1$. The widths of the bumps are approximately the same as in the case of no drift, though the drift is more significant in case when $\:{\epsilon}_{\eta\:}={\epsilon}_{\zeta\:}=0.005$ than when $\:{\epsilon}_{\eta\:}={\epsilon}_{\zeta\:}=0.01$. This is expected as the run time is 4x longer in the first case.

**Fig. 4:**
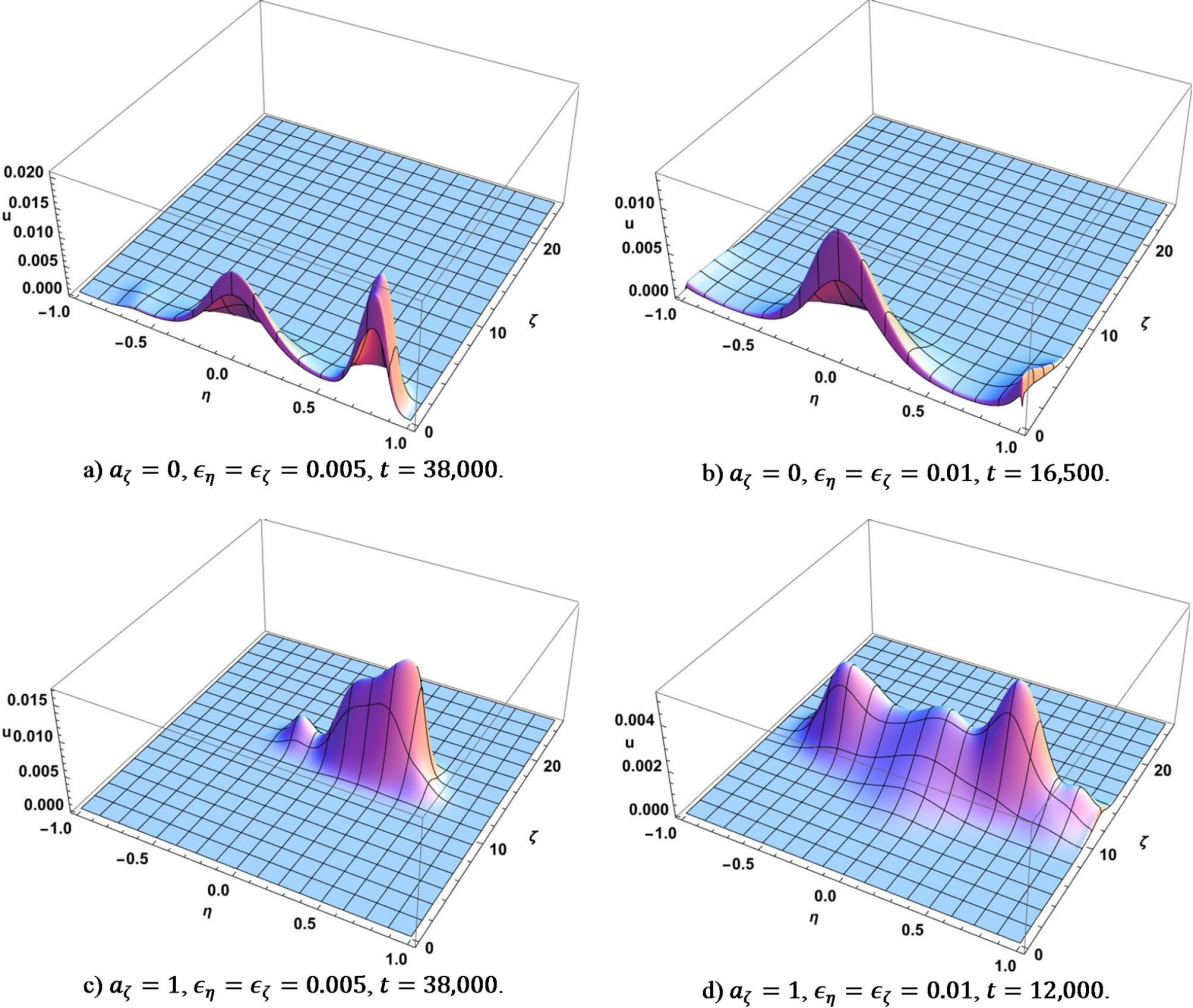
Dependence of $\:U$ on $\:\eta\:$ and $\:\zeta\:$ for some values of time when the system experiences rapid transformations. Produced using Wolfram Mathematica 13.

Figure 4 shows some points in time for the four considered scenarios where each of the corresponding diffusion-like evolution runs break down and experience rapid transformation. We will talk about that in more detail when we discuss Fig. 2. Here we would like to note that the process is essentially random and using different random seed values will produce different intermediate shapes until the process settles down near stationary points.

**Fig. 5:**
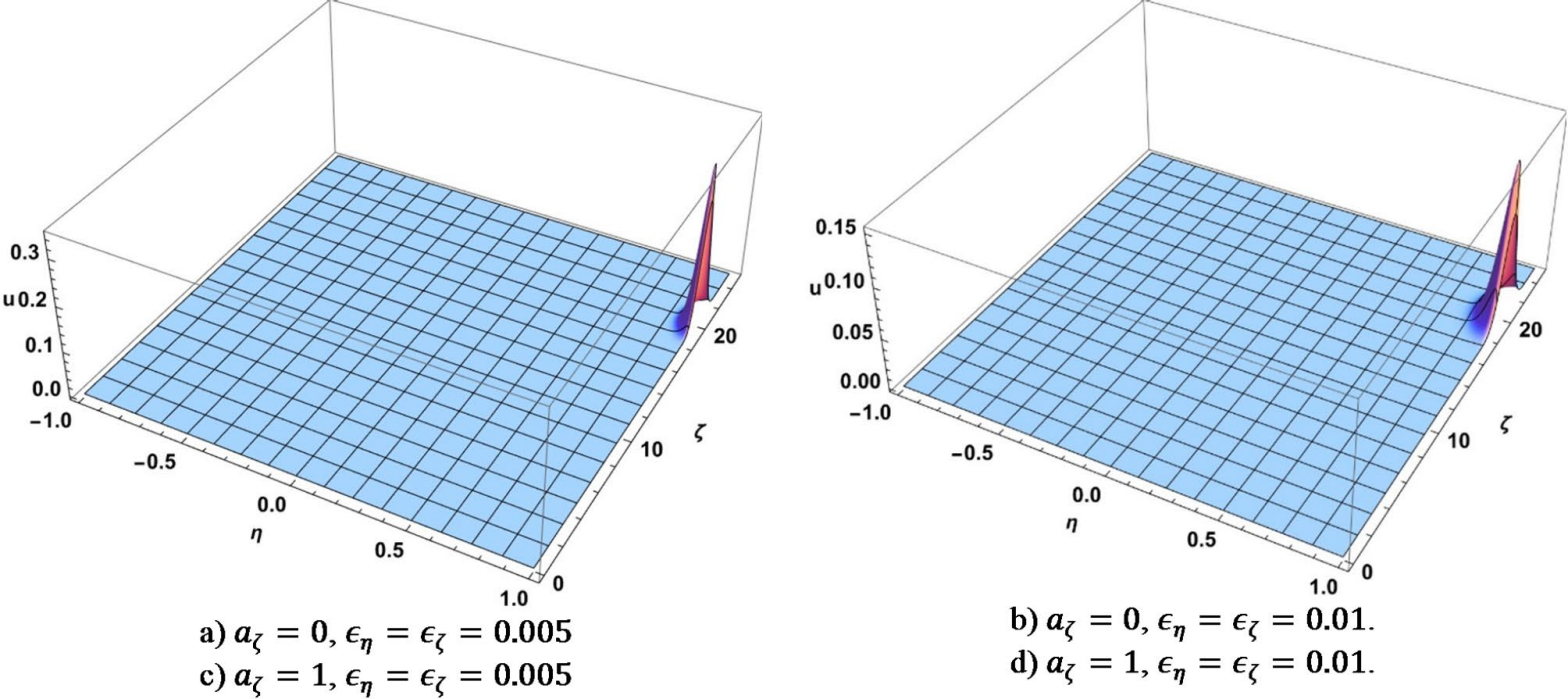
Dependence of $\:U$ on $\:\eta\:$ and $\:\zeta\:$ near stationary point ($\:t=\text{200,000}$). Produced using Wolfram Mathematica 13.

Figure 5 shows the point in time ($\:t=\text{200,000}$) when the stationary point has been reached. The stationary point looks nearly the same for all considered variants: it is a narrow peak near $\:\eta\:\approx\:1$ and $\:{\zeta\:}_{max}\approx\:20\div 21$, which is a point in $\:\zeta\:$ space where $\:\gamma\:\left(\eta\:,\zeta\:\right)$ experiences a rapid increase due to a limiting factor $\:f$. Without that limiting factor the system would just run toward $\:\zeta\:=L$. The stationary point for $\:{\epsilon}_{\eta\:}={\epsilon}_{\zeta\:}=0.005$ is a narrower peak than for $\:{\epsilon}_{\eta\:}={\epsilon}_{\zeta\:}=0.01$, as expected, and the value of $\:{a}_{\zeta\:}$ does not affect the position and the width of the peak.

We present animation of the evolution of the system for these 4 scenarios in supplementary materials.

We also want to look at some cumulative characteristics of the evolution because it is hard to present changes to a 3D chart over time in a compact form. Means and standard deviations of $\:U$ in $\:\eta\:$ and $\:\zeta\:$ spaces are a convenient choice. In integral representation they can be expressed as follows (note that the normalization here is different in comparison to Eq. (9)):

39 $$\begin{aligned}{U}_{0}\left(t\right)&=\underset{-1}{\overset{1}{\int\:}}\underset{0}{\overset{L}{\int\:}}U\left({\eta\:}^{{\prime\:}},{\zeta\:}^{{\prime\:}},t\right)\:d{\eta\:}^{{\prime\:}}\:d{\zeta\:}^{{\prime\:}},\:u\left({\eta\:}^{{\prime\:}},{\zeta\:}^{{\prime\:}},t\right)\nonumber\\&\equiv\:\frac{U\left({\eta\:}^{{\prime\:}},{\zeta\:}^{{\prime\:}},t\right)}{{U}_{0}\left(t\right)}\:{\mu\:}_{\eta\:}=\underset{-1}{\overset{1}{\int\:}}\underset{0}{\overset{L}{\int\:}}{\eta\:}^{{\prime\:}}\:u\left({\eta\:}^{{\prime\:}},{\zeta\:}^{{\prime\:}},t\right)\:d{\eta\:}^{{\prime\:}}\:d{\zeta\:}^{{\prime\:}},\:{\mu\:}_{\zeta\:}\nonumber\\&=\underset{-1}{\overset{1}{\int\:}}\underset{0}{\overset{L}{\int\:}}{\zeta\:}^{{\prime\:}}\:u\left({\eta\:}^{{\prime\:}},{\zeta\:}^{{\prime\:}},t\right)\:d{\eta\:}^{{\prime\:}}\:d{\zeta\:}^{{\prime\:}}{\sigma\:}_{\eta\:}^{2}\nonumber\\&=\underset{-1}{\overset{1}{\int\:}}\underset{0}{\overset{L}{\int\:}}{\left({\eta\:}^{{\prime\:}}-{\mu\:}_{\eta\:}\right)}^{2}\:u\left({\eta\:}^{{\prime\:}},{\zeta\:}^{{\prime\:}},t\right)\:d{\eta\:}^{{\prime\:}}\:d{\zeta\:}^{{\prime\:}},\:{\sigma\:}_{\zeta\:}^{2}\nonumber\\&=\underset{-1}{\overset{1}{\int\:}}\underset{0}{\overset{L}{\int\:}}{\left({\zeta\:}^{{\prime\:}}-{\mu\:}_{\zeta\:}\right)}^{2}\:u\left({\eta\:}^{{\prime\:}},{\zeta\:}^{{\prime\:}},t\right)\:d{\eta\:}^{{\prime\:}}\:d{\zeta\:}^{{\prime\:}}\end{aligned}$$

**Fig. 6:**
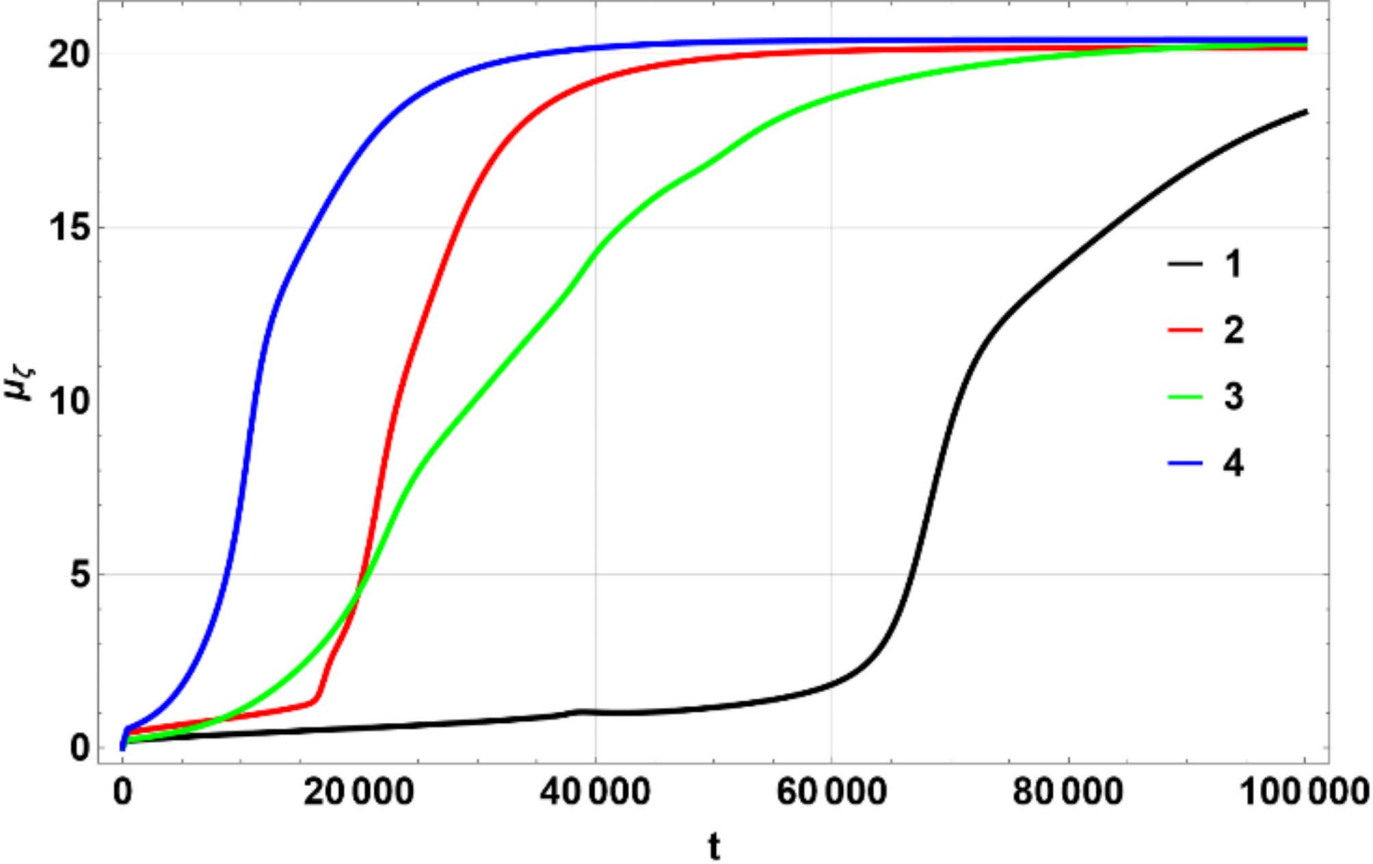
Dependence of $\:{\mu\:}_{\zeta\:}$ on $\:t$. Produced using Wolfram Mathematica 13.

**Fig. 7:**
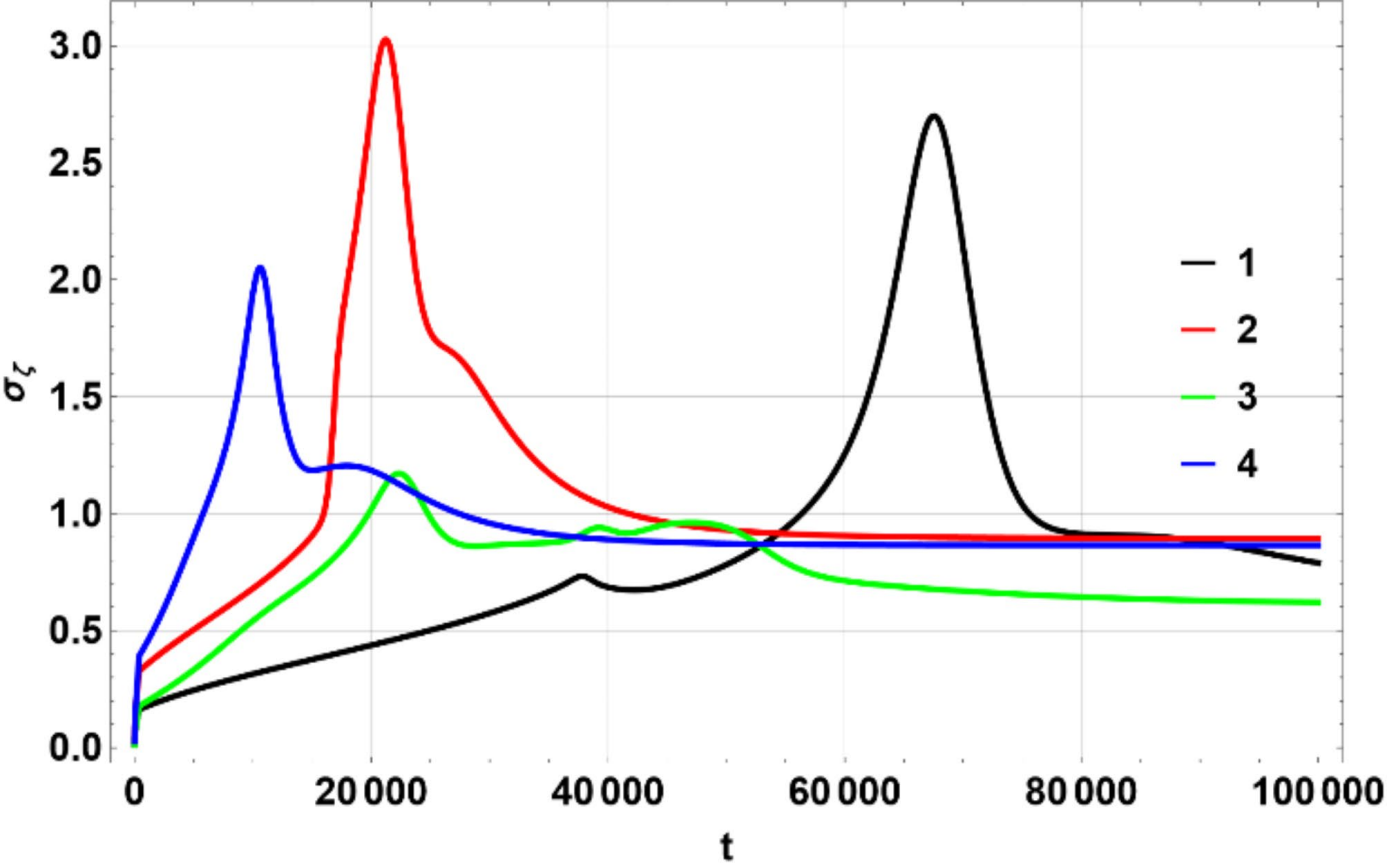
Dependence of $\:{\sigma\:}_{\zeta\:}$ on $\:t$. Produced using Wolfram Mathematica 13.

Figures 1, 2 and 6, and 7 show $\:{\mu\:}_{\eta\:}$, $\:{\sigma\:}_{\eta\:}$, $\:{\mu\:}_{\zeta\:}$, $\:{\sigma\:}_{\zeta\:}$ for the four scenarios considered above. All figures have lines 1 through 4 corresponding to the parameters shown in the Table 1 above.

The results presented above show that a two-dimensional model is much more complex and richer than a one-dimensional one^23^. In particular, as food becomes scarce, the boundary between efficient and inefficient protocells is just two points in one-dimensional model. And so, these points are not connected. That boundary becomes a connected curve in the two-dimensional model. Those protocells that are inside the boundary “die off” from starvation because they are not efficient enough to sustain their population at a given concentration of food. And the boundary extends from $\:\left(0,\:0\right)$ over time due to diffusion and appearance of more efficient species. The most interesting part here is that many species are possible. Second, random fluctuations may create several favorable mutations in a row. A favorable mutation is a mutation that makes some species of protocells more efficient. That slight increase in efficiency results in placing some small number of protocells into the place in $\:\left(\eta\:,\zeta\:\right)$ space where they have a positive exponential growth factor in comparison to the boundary where the bulk of the protocells exist at some moment in time. Subsequently these more efficient protocells can temporarily experience exponential growth. This may result in splitting of the original “bump” distribution of protocells into several non-connected groups, which we can call species. The greater the total number of molecules in the system the more likely this is to occur.

The easiest way to illustrate four stages of evolution of the system is to look at the concentration of food molecules. As the time scale of the first stage is much faster than that of the other three stages, we need two charts to showcase all four stages.

**Fig. 8:**
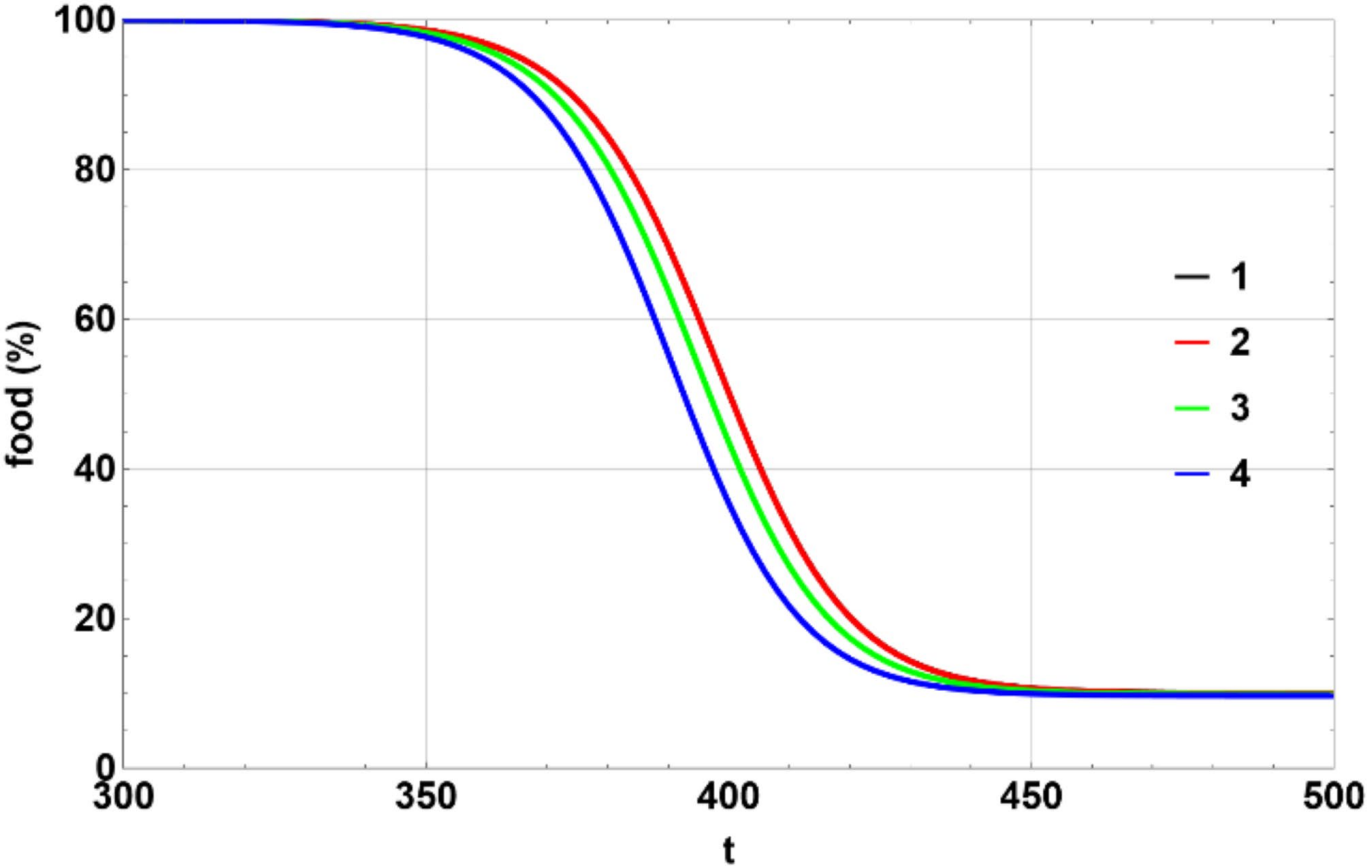
Dependence of the amount of food in percent on $\:t$ during stage 1. Produced using Wolfram Mathematica 13.

Figure 8 illustrates the first stage of evolution. It shows an exponential, or more correctly, a hyperbolic tangent-like shape. Models 1 and 2 are indistinguishable from each other during this stage (that’s why model 1’s chart is not visible in the figure). Model 3 is slightly faster, and model 4 is even faster at consuming most of the food. Models 3 and 4 have a drift coefficient, which moves protocells into the region of higher efficiency (resulting in faster food consumption). Model 4 also has a larger diffusion coefficient (larger values of $\:{\epsilon}_{\eta\:}$ and $\:{\epsilon}_{\zeta\:}$), which further accelerates some protocells into the region of higher efficiency.

**Fig. 9:**
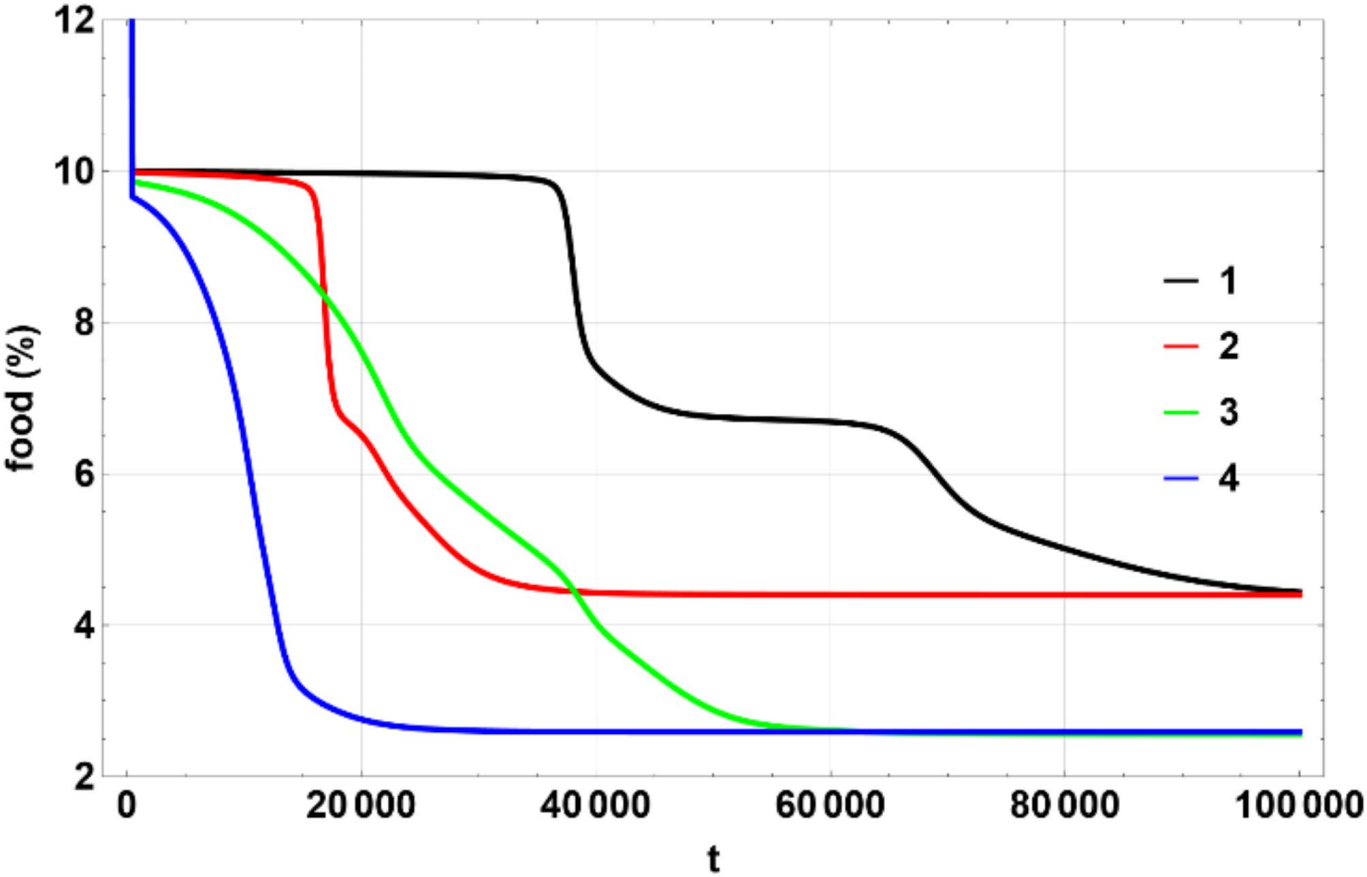
Dependence of the amount of food in percent on $\:t$ during stages 2 – 4. Produced using Wolfram Mathematica 13.

Figure 9 illustrates stages 2–4 of the evolution. Of all the models considered here, only model 1 shows four clear stages of evolution, where stages 2 and 3 appear as temporary plateaus, followed by a sharp transition to the next stage. Model 2, which has a zero-drift coefficient like model 1 but a larger diffusion coefficient, also exhibits a clear stage 2, while stages 3 and 4 are much faster and not clearly visible. Model 3 has a drift coefficient but a smaller diffusion coefficient. The drift coefficient moves the protocells into the region of higher efficiency, making the plateaus almost disappear. Finally, model 4 has both a drift coefficient and a larger diffusion coefficient, combining stages 2–4 into a single continuous slide.

## Conclusions

We consider an evolution of protocells without competition in a two-dimensional space: total enantioselectivity, $\:\eta\:$, and the amount of information passed from generation to generation, $\:\zeta\:$, and we present a simple two-dimensional model, which describes such an evolution. We show that such a model is described by a system of integrodifferential equations, which under the hood is powered by a protocell replication kernel, $\:K\left(\eta\:,\zeta\:,{\eta\:}^{{\prime\:}},{\zeta\:}^{{\prime\:}}\right)$, and a mortality rate, $\:\gamma\:\left(\eta\:,\zeta\:\right)$. We show that it is convenient to split a replication kernel into a multiplication of the pieces: a total replication rate at a point $\:\left(\text{0,0}\right)$ in $\:\left(\eta\:,\zeta\:\right)$ space, $\:{k}_{0}$, a total normalized replication rate, $\:{k}_{a}\left({\eta\:}^{{\prime\:}},{\zeta\:}^{{\prime\:}}\right)$, which is the normalized rate at which the species with total enantioselectivity $\:{\eta\:}^{{\prime\:}}$ and the amount of stored information $\:{\zeta\:}^{{\prime\:}}$ can produce any species, and the probability that species with total enantiomeric excess $\:{\eta\:}^{{\prime\:}}$ and amount of stored information $\:{\zeta\:}^{{\prime\:}}$ would produce species with total enantiomeric excess $\:\eta\:$ and amount of stored information $\:\zeta\:$, $\:p\left(\eta\:,\zeta\:,{\eta\:}^{{\prime\:}},{\zeta\:}^{{\prime\:}}\right)$.

We show that observation of life, as it exists, leads to a conclusion that a Taylor expansion of total normalized replication rate $\:{k}_{a}\left(\eta\:,\zeta\:\right)$ near point $\:\left(0,0\right)$ should be a function with zero linear coefficient in $\:\eta\:$ space, non-negative linear coefficient in $\:\zeta\:$ space, and positive quadratic coefficients both in $\:\eta\:$ and $\:\zeta\:$ spaces, provided that we limit the considerations to the terms not higher than quadratic ones.

We show that under reasonable assumptions the mutations from generation to generation of protocells are small (the probability $\:p\left(\eta\:,\zeta\:,{\eta\:}^{{\prime\:}},{\zeta\:}^{{\prime\:}}\right)$ is a narrow peak centered near $\:\eta\:={\eta\:}^{{\prime\:}}$ and $\:\zeta\:={\zeta\:}^{{\prime\:}}$), the evolution of the system can be described in four stages.

The first stage is a very quick exponential growth of the population of the nearly racemic species near the point $\:\left(\text{0,0}\right)$ in $\:\left(\eta\:,\zeta\:\right)$ space. Since this is an exponential process, it should happen in a negligible amount time on a geological scale, and we show that the time estimate for that process is on the order of $\:{t}_{0}<{10}^{2}$ years for the closed model considered here.

The second stage is a relatively slow diffusion-like process, when the system spreads out in both $\:\eta\:$ and $\:\zeta\:$ directions. The system at that stage can be very well approximated by a convection-diffusion equation with a drift. If a linear coefficient in the Taylor expansion of total normalized replication rate $\:{k}_{a}\left(\eta\:,\zeta\:\right)$ near point $\:\left(0,0\right)$ in $\:\zeta\:$ space is zero or small enough, then the system experiences just a diffusion (spearing out of the initial delta function like peak), and if that linear coefficient is large enough then that peak travels in $\:\zeta\:$ space while spreading out, thus, gaining information storage capacity able to be passed from generation to generation. The time of that stage depends on the combination of factors: larger mutation rates in $\:p\left(\eta\:,\zeta\:,{\eta\:}^{{\prime\:}},{\zeta\:}^{{\prime\:}}\right)$ and larger quadratic coefficients in $\:{k}_{a}\left(\eta\:,\zeta\:\right)$ make it faster and the other way around.

The third stage starts when diffusion powered widening of the initial narrow peak slows down enough in comparison to local drift at the edges of the peak. That’s the point in time when the process becomes stochastic in nature. One or several “species” could be formed, and they run away in the direction of increased efficiency of the species. This process is quick as it is also exponential in nature, though not as quick as the first stage. It is exponential because a small number of more efficient protocells find the food abundant whereas the bulk of the protocells balance between replication and extinction. When the grid step is sufficiently small to resolve a global asymmetry factor, the system consistently produces more efficient species. As we used a grid with $\:d=500$ points in both $\:\left(\eta\:,\zeta\:\right)$ directions, that corresponds to global asymmetry factor $\:g\sim\frac{1}{d}=0.002$. We performed multiple runs, and the results consistently show directed symmetry breaking in the system. Smaller values of $\:g$ may result in two species form near $\:\eta\:\approx\:\pm\:1$, and less efficient species ($\:\eta\:\approx\:-1$ in our case) may dominate in the system for some prolonged period, after which more efficient species may still overcome less efficient ones. If the total amount of food in the system $\:{\rho\:}_{0}$ is larger, then such event is more likely to happen. This is because for this event to occur there must be at least some protocells near $\:\eta\:\approx\:-1$ and some large enough value of $\:\zeta\:$. However, if $\:{\rho\:}_{0}$ is small, thenе this may not statistically happen and symmetry breaking becomes random when a grid cannot resolve the global asymmetry factor.

The fourth stage is a relaxation toward a stationary point, which is a narrow peak near $\:\eta\:\approx\:1$, $\:{\zeta\:}_{max}\approx\:20 \div 21$ in case of the models considered here. When $\:{a}_{\zeta\:}=0$, then symmetry breaking occurs first and the system transitions toward a peak $\:\eta\:\approx\:1$ and some small value of $\:\zeta\:$ first, then slides toward $\:{\zeta\:}_{max}$ either as a whole bump or forming some runaway species. When $\:{a}_{\zeta\:}$ is large enough (we considered $\:{a}_{\zeta\:}=1$ and it is large enough for the model considered here), then the system first accumulates substantial ability to pass information from generation to generation without experiencing any symmetry breaking, and symmetry breaking occurs only when the system already possesses substantial ability to pass information from generation to generation.

## Electronic supplementary material

Below is the link to the electronic supplementary material.

Supplementary Material 1

Supplementary Material 2

Supplementary Material 3

Supplementary Material 4

Supplementary Material 5

Supplementary Material 6

## Data Availability

The datasets generated during the current study are available in a “frozen” GitHub repository branch: https://github.com/kkkmail/CoreClm/tree/clm700-Fredholm-frozen-V2.
