# Supplementary material for: Chiral symmetry breaking and information accumulation in pre-biological protocell evolution
Source: Sci Rep. 2025 Apr 14;15:12806. doi: 10.1038/s41598-025-97319-2 (PMC11997073; doi:10.1038/s41598-025-97319-2)
Supplement: Supplementary file 5 — Supplementary Material 5 [file 41598_2025_97319_MOESM5_ESM.docx]

# **Legends for the supplementary videos**

**d500k1e005g01a002f1E_200K__V2.mp4**

Time evolution of $U\left( \eta,\zeta,t \right)$ for: $b_{\eta}=1$, $a_{\zeta}=0$, $b_{\zeta}=1$, $\epsilon_{\eta}=\epsilon_{\zeta}=0.005$, $g=0.002$, $\rho_{0}={10}^{18}$, $t_{\max}=200K$.

**d500k1e005g01a002i10f1E_200K__V2.mp4**

Time evolution of $U\left( \eta,\zeta,t \right)$ for: $b_{\eta}=1$, $a_{\zeta}=0$, $b_{\zeta}=1$, $\epsilon_{\eta}=\epsilon_{\zeta}=0.01$, $g=0.002$, $\rho_{0}={10}^{18}$, $t_{\max}=200K$.

**d500k1e01g01a002f1E_200K__V2.mp4**

Time evolution of $U\left( \eta,\zeta,t \right)$ for: $b_{\eta}=1$, $a_{\zeta}=1$, $b_{\zeta}=1$, $\epsilon_{\eta}=\epsilon_{\zeta}=0.005$, $g=0.002$, $\rho_{0}={10}^{18}$, $t_{\max}=200K$.

**d500k1e01g01a002i10f1E_200K__V2.mp4**

Time evolution of $U\left( \eta,\zeta,t \right)$ for: $b_{\eta}=1$, $a_{\zeta}=1$, $b_{\zeta}=1$, $\epsilon_{\eta}=\epsilon_{\zeta}=0.01$, $g=0.002$, $\rho_{0}={10}^{18}$, $t_{\max}=200K$.
