# Supplementary material for: Chiral symmetry breaking and information accumulation in pre-biological protocell evolution
Source: Sci Rep. 2025 Apr 14;15:12806. doi: 10.1038/s41598-025-97319-2 (PMC11997073; doi:10.1038/s41598-025-97319-2)
Supplement: Supplementary file 6 — Supplementary Material 6 [file 41598_2025_97319_MOESM6_ESM.docx]

# **Description of the Models and Content of the Repository**

The datasets generated during the current study are available in a “frozen” GitHub repository branch: <https://github.com/kkkmail/CoreClm/tree/clm700-Fredholm-frozen-V2>. The models used in the current research are described in Table 1 in the manuscript and carry the following internal model codes:

1. d500k1e005g01a002f1E
2. d500k1e01g01a002f1E
3. d500k1e005g01a002i10f1E
4. d500k1e01g01a002i10f1E

The models can be examined in a “frozen” GitHub repository branch referenced above. The model runners are located in <https://github.com/kkkmail/CoreClm/blob/clm700-Fredholm-frozen-V2/CoreClm/FredholmSolverTests/PoissonTests.fs> file with the names of the methods starting from model codes above. All code is written in F# and the code runs on Windows.

Further description assumes that the branch is pulled into the **C:\GitHub\CoreClm** folder on Windows and that the **C:\EeInf** folder exists.

Each model produces multiple Wolfram Mathematica files with **m** extension when it is run. The file names consist of a prefix, which follows the pattern **ModelName_RunTime__DateStamp_TimeStamp**, e.g. **d500k1e01g01a002f1E_200K__20240310_****1407**, where the **ModelName** (**d500k1e01g01a002f1E**) is the name of the model, **RunTime** is the number of evolution steps (e.g., **200K** means 200,000 steps), **DateStamp** (**20240310**) are the year, month, and day when the model was started, **TimeStamp** (**1407**) is the hour and minute when the model was started in 24 hour format, and some optional part. We will use the prefix above as an example. The main file (about 106 MB) is **d500k1e01g01a002f1E_200K__20240310_1407.m**. It contains full model data, some aggregate values over time, which can be used to generate aggregate time evolution charts (like means and standard deviations), a final state of the system, and a generated call to some Mathematica function to perform evaluation of the data in that module. This file will be placed into **C:\EeInf** folder. That file will produce some aggregate charts, when run in Mathematica. The model run will also produce frame files to assemble animation of system evolution. The main animation file **d500k1e01g01a002f1E_200K__20240310_1407__animation.m** is placed into the same folder. It contains a call to produce an animation of the system evolution. Each frame is first exported into an **m** data file with the additional numerical suffix, which is a number of the evolution step zero padded up to 8 characters, like: **d500k1e01g01a002f1E_200K__20240310_1407__00000000.m** during the original model run. Each frame data file is about 13 MB. When the animation module is run, it will produce a PNG picture of each frame, which are then assembled into a unified animation using FFMPEG.

All charts in the manuscript were generated using Wolfram Mathematica 13: <https://www.wolfram.com/mathematica/>. Two-dimensional charts (1 – 3) were generated in two steps. First, all data frames (as **m** data files) were produced during system runs, as described above. Then all PNG animation frames of all models were generated using the file <https://github.com/kkkmail/CoreClm/blob/clm700-Fredholm-frozen-V2/Math/CreateAllAnimationsQuick.nb>, which processes all found ***__animation.m** files. Some frames were then chosen for the publication. One-dimensional charts (4 – 9) were generated using the file <https://github.com/kkkmail/CoreClm/blob/clm700-Fredholm-frozen-V2/Math/mean_stdDev__001.nb>. In order to run this Mathematica notebook, the archive located at <https://github.com/kkkmail/CoreClm/blob/clm700-Fredholm-frozen-V2/Math/allData__200K.rar> must be unpacked into the folder where it is located.
